# Effects of vibrotactile feedback on yoga practice

**DOI:** 10.3389/fspor.2022.1005003

**Published:** 2022-10-31

**Authors:** Md Shafiqul Islam, Sang Won Lee, Samantha M. Harden, Sol Lim

**Affiliations:** ^1^Department of Industrial and Systems Engineering, Virginia Tech, Blacksburg, VA, United States; ^2^Department of Computer Science, Virginia Tech, Blacksburg, VA, United States; ^3^Department of Human Nutrition, Foods, and Exercise, Virginia Tech, Blacksburg, VA, United States

**Keywords:** remote physical activity, vibrotactile feedback, yoga, vision impairments, regular exercise

## Abstract

Participating in physical exercise using remote platforms is challenging for people with vision impairment due to their lack of vision. Thus, there is a need to provide nonvisual feedback to this population to improve the performance and safety of remote exercise. In this study, the effects of different nonvisual types of feedback (verbal, vibrotactile, and combined verbal and vibrotactile) for movement correction were tested with 22 participants with normal vision to investigate the feasibility of the feedback system and pilot tested with four participants with impaired vision. The study with normal-vision participants found that nonvisual feedback successfully corrected an additional 11.2% of movements compared to the no-feedback condition. Vibrotactile feedback was the most time-efficient among other types of feedback in correcting poses. Participants with normal vision rated multimodal feedback as the most strongly preferred modality. In a pilot test, participants with impaired vision also showed a similar trend. Overall, the study found providing vibrotactile (or multimodal) feedback during physical exercise to be an effective way of improving exercise performance. Implications for future training platform development with vibrotactile or multimodal feedback for people with impaired vision are discussed.

## 1. Introduction

Regular physical activity improves quality of life by enhancing both physical and mental well-being (1–3). Evidence exists to support the positive effects of regular physical activity in mitigating health-related risks, such as cardiovascular diseases (4) and functional loss (5), as well as improving cognitive functioning (6). It also helps to shorten the period of disability preceding the end of life (7). Despite physical activity having proven positive effects on physical and mental health, barriers prevent people with vision impairment (VI) from actively participating in exercise programs (8, 9). Traditional barriers hindering active participation among people with VI include lack of accessibility (e.g., transportation) (10), affordability (11), fear of injury (11), and inadequate support (12). The COVID-19 pandemic has also led to an increase in physical inactivity among these populations (13). Due to these challenges, there are low levels of participation in physical activity, poorer physical fitness, and high prevalence of obesity among this group (14). An exercise system that caters to the needs of this group and motivates its members to actively engage in regular physical exercise while removing barriers is needed to mitigate the adverse effects of a sedentary lifestyle.

As a potential solution to these barriers, and to increase participation and engagement in physical activity among people with VI, technology-based remote exercise programs are increasingly used for independent exercise at home (15). These programs can offer a solution to the lack of accessibility, especially the need for transportation, as people can participate and engage in physical activity from home without the need to visit a facility in person (16, 17). Overall, the remote virtual fitness market is expected to grow by 33.1% per year, becoming a $59.2 billion industry by the year 2027 (18). Examples of remote exercise platforms targeting VI groups include sensor-based commercial exergames [e.g., Nintendo's Wii Sports, Ring Fit Adventure, and Wii Fit; (19, 20)], as well as custom exergames like VI-bowling (21), VI-tennis (15), and Eyes-free yoga (22). These exergame platforms designed for VI use verbal feedback unimodally (22) or combine verbal and vibrotactile feedback multimodally (15, 21). However, the vibrotactile feedback in the studies cited here used only one tactor to transmit vibration to one body part (e.g., the dominant arm), thus limiting it to providing simple guidance (e.g., pass/fail grading).

Besides those exergames designed specifically for VI groups, existing remote exercise learning platforms commonly provide feedback on users' performance through visual and auditory prompts. These commonly provided modalities may be either useless (e.g., visual) may be either useless or insufficient for those with impaired vision during remote physical activity (23). Without a feasible alternative to compensate for their lack of vision, performing and learning exercise using remote platforms can be challenging, unsafe, and unsustainable. Therefore, there is a need to deliver feedback through nonvisual sensory channels (e.g., verbal, vibrotactile, force, or a combination thereof) to provide effective and easily understood communication for motor learning among the VI population (24, 25). Notably, previous research has explored the benefits of using real-time vibrotactile feedback to effectively deliver localized tactile cues to learners (26, 27) and provide intervention to improve sensorimotor performance (28, 29). The use of vibrotactile feedback in combination with other sensory feedback modalities has been proven to enhance motor learning ability, including when learning a new movement or reviewing a previously learned one (26, 30, 31), as well as when learning, guiding, and practicing physical activities (29, 32, 33). Nonvisual multimodal feedback has also been utilized to enhance motor learning experiences and performance specifically for people with VI (34). Multimodal feedback combining vibrotactile feedback with other feedback modalities (i.e., visual and auditory) is known to yield better performance results [e.g., faster reaction time; (35)] compared to unimodal feedback (25, 36). Despite the benefits of and growing interest in using multimodal sensory feedback in remote physical training (37, 38), the effectiveness of vibrotactile feedback on people with VI in the context of technology-based remote exercise systems (i.e., exercise over video-conferencing tools, telerehabilitation) is still underexplored (21).

To test the effectiveness of a multimodal feedback system in remote physical training for people with VI, we selected yoga as the physical activity for this study. Yoga has shown potential health and accessibility benefits as a remote exercise option (39). Specifically, yoga directly benefits numerous physical abilities: balance, flexibility, motor coordination, strength, and cardiovascular endurance (40–44). It also improves overall quality of life (45) while reducing stress (46, 47) and anxiety (48). Practicing yoga regularly promotes healthy aging as it improves stability and mobility (49). Also, yoga does not require special equipment, can be performed indoors, and is adaptable to each individual's physical capabilities through pose modifications and variations. Its accessibility and flexibility make yoga a suitable form of remote physical exercise for people with VI (22, 50) as well as broader populations, including people with disabilities (51), chronic pain (52), and Parkinson's disease (53), and older adults with preexisting health conditions (54). In addition, we found yoga to be suitable for our study because a yoga session is often composed of a mixture of static poses with dynamic transitions which are relatively slow-paced; thus, perceiving sensory feedback with a vibrotactile feedback system is more feasible when performing yoga compared to performing a more fast-paced activity.

The objective of this study is to investigate the effects of using vibrotactile and multimodal feedback in a physical, pose-based practice (*asana* in Sanskrit) as a means of guiding a remote yoga practice for people with VI. We investigated the following three hypotheses to test the effects of vibrotactile feedback in a session of yoga as exercise:

*H*_1_(effectiveness): Vibrotactile or multimodal (vibrotactile + verbal) feedback is more effective than no feedback or verbal feedback.
*H*_2_(time-efficiency): Vibrotactile feedback is more time-efficient than verbal feedback.
*H*_3_(user preference ratings): Users prefer any type of nonvisual feedback over no feedback at all.

We performed two separate preliminary studies with people with no vision impairment (NVI) to test our overall feedback system, and one follow-up pilot study with people with vision impairment (VI). The goal of our study with NVI subjects was to conduct an initial assessment of the feedback system's effectiveness and to check the system's feasibility and safety before pilot-testing the system with the target population of interest (VI).

## 2. Methods

### 2.1. Study participants

Twenty-two NVI participants (9 males and 13 females, age: 27.6 ± 13.0 years) were recruited from the local community. Participants were included in the study if they were 18 years or older, were able to walk and stand without pain and discomfort, and were novice yogis (i.e., had not participated in yoga more than 10 times in the past 5 years). All participants reported no back pain in the 12 months prior to the study, and all were able to walk short distances without pain. Participants self-reported their confidence in balance using the Activities-Specific Balance Confidence (ABC) Scale (55). All participants provided written informed consent prior to participation, following the procedures approved by the University's Institutional Review Board.

### 2.2. Overview of yoga learning trials, feedback system, and study procedures

#### 2.2.1. Modular learning scenarios with step-by-step instructions

To increase the validity of the evaluation, realistic yoga learning scenarios involving yoga poses of varying difficulty were constructed according to rigorous task analyses and pose difficulty ratings from instructors. A typical yoga practice (30–60 min) consists of a sequence of yoga poses that progresses toward more challenging poses; similar poses are grouped together, and participants may explore pose modifications and variations within the sequence. Additionally, participants arrive at and leave each yoga pose (static) through multiple movement steps (dynamic transition between poses). To create such learning scenarios for the study, hierarchical task analyses (HTAs, 56) were performed based on a preliminary study (50). For the purpose of this study, the three most commonly performed starting poses; namely, Standing (Mountain) Pose, Table Pose, and Staff Pose, were selected for the HTA analyses, each with 15 pose variations stemming from the starting pose (see Supplementary Figures 1–3). The HTA and selection of yoga poses were based on an extensive review of instructional manuals and books (57–59), as well as instructional videos (e.g., from https://yogawithadriene.com). From the HTA, each yoga pose was broken down into movement steps wherein the learner changes only one body segment or joint (or a pair of segments or joints) at a time. A connection was drawn between different movement steps “descended” from a single starting pose when movement steps between poses overlapped (see arrows in Supplementary Figures 4–6). The decomposed movement steps and associated verbal instructions were pilot-tested with people who had never practiced yoga before and revised recursively to avoid ambiguity. A more detailed description of how the HTA was performed can be found in a prior study (50). The final hierarchical structures representing the outcome of the HTA, including the pose names, step-by-step verbal instructions for movement steps, and their connections are depicted in Supplementary Figures (Supplementary Figures 1, 4: Standing Pose, Supplementary Figures 2, 5: Table Pose, and Supplementary Figures 3, 6: Staff Pose).

#### 2.2.2. Feedback system

Four different feedback conditions were tested in this study; namely, no feedback (NF), verbal feedback (Ver), vibrotactile feedback (Vib), and multimodal feedback with both verbal and vibrotactile feedback (Ver+Vib). Visual demonstrations and feedback were not provided in the study to simulate a condition in which pose instructions are delivered purely in a step-by-step verbal format, targeting those with VI as main users of the system. This also simulates a condition for NVI users in which visual demonstrations and feedback, while present, are difficult to see due to either the size of the screen used for the session or the type of pose being performed, increasing the difficulty and ambiguity of the activity. For example, many poses grouped beneath the Table Pose, such as the down dog split pose (Pose 2.4 in Supplementary Figure 2), require participants to face downwards during the practice. To keep an eye on visual demonstrations and feedback, participants might be forced to twist their neck or torso sideways or upwards, creating unnecessary strain and potentially leading to injury. Thus, our intention was to create a platform similar to “Eyes-Free Yoga,” (60) which was designed to allow people with VI to practice yoga without visual demonstrations, but for a broader population that also includes NVI considering practical implications of remote exercise learning scenarios.

To complete each pose (represented by a rounded box in Supplementary Figures), multiple sequential movement steps (shown as blocks in Supplementary Figures) must be correctly performed. For example, Tree Pose (1.1) is completed when all the movement steps between Pose 1.0 and Pose 1.1 (i.e., 1.1–1, 1.1–2, 1.1–3, and 1.1–4 in Supplementary Figure 4) are correctly performed. In this study, feedback was given at the end of each step (block) by assessing the correctness of the pose following a verbal instruction.

**Figure 1:**
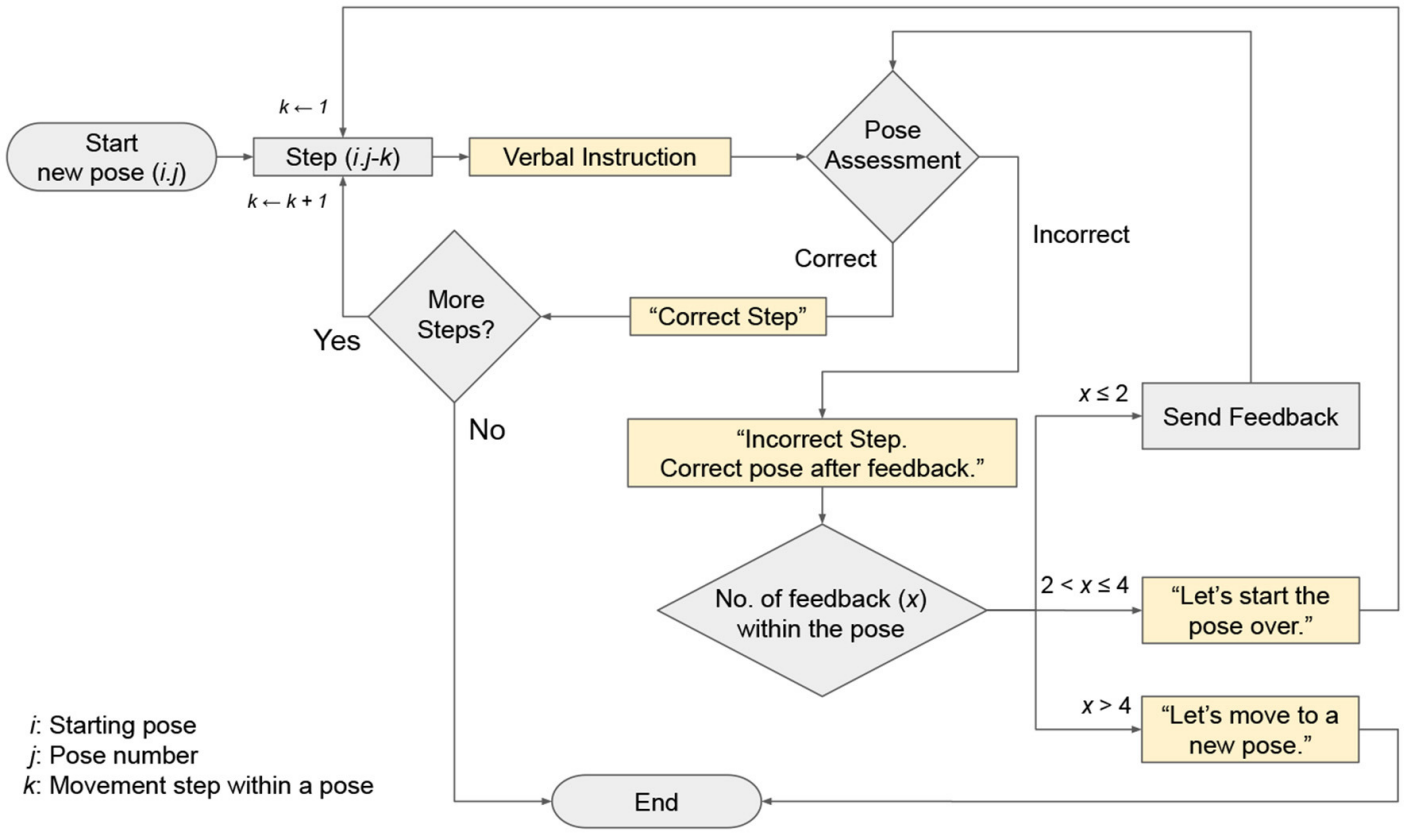
Sequence of decisions made in system for sending verbal notifications and feedback during yoga.

**Figure 2:**
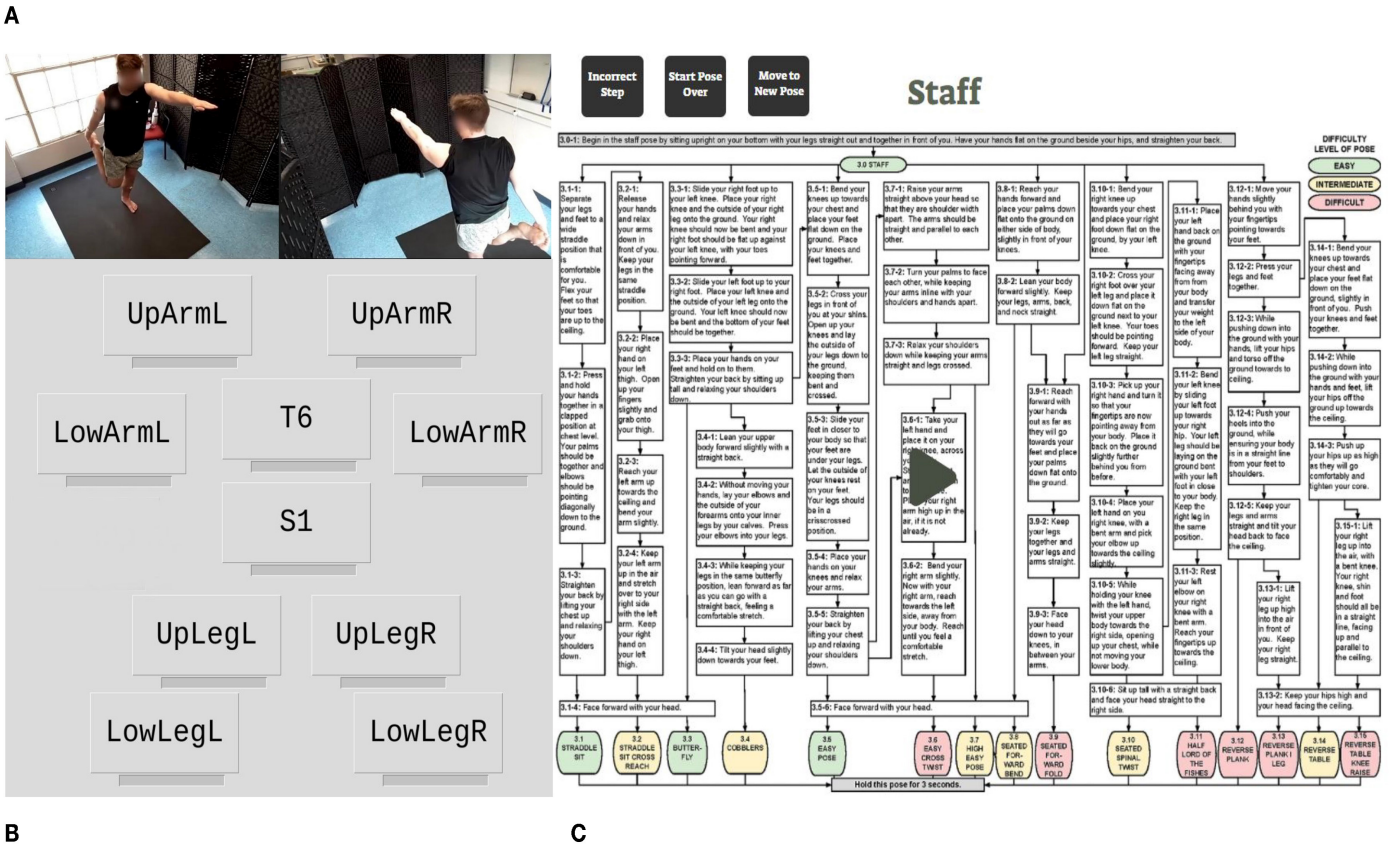
A custom web-based application developed for the feedback system. **(A)** Two camera views (front and back). **(B)** Interface for sending vibrotactile feedback. **(C)** Interface for sending verbal instructions and feedback.

Figure 1 presents a flowchart of the feedback system developed for the study. Within a pose (*i*.*j*, where *j* is a pose variation number under the staring pose, *i*), participants were asked to follow a verbal instruction (*i*.*j*–*k*) that was used to guide them into a correct movement step. For example, 1.2–2 indicates the second verbal instruction needed to transition from Standing (Pose 1.0) to Clasped Eagle (Pose 1.2): “*Bend your left knee significantly so that your body is lowered”* in Supplementary Figure 4. In each step, the participant's pose was assessed visually by a trained researcher using a real-time video feed from two cameras installed to capture the front and back of the participant's full body posture (Figure 2A). A custom web-based application was developed to send different types of sensory feedback through a speaker (for Ver feedback, Figure 2C), wearable sensors (for Vib feedback, Figure 2B), or both (for Ver+Vib feedback) remotely after a researcher has assessed the pose and triggered a feedback event by choosing a feedback type and location. If the participant's postural angle was found to deviate from the correct pose by more than 30° according to a visual inspection by our trained researcher, the participant received a verbal notification: “Incorrect step. Correct pose after the feedback.” If the step was correct, a verbal notification indicating their correctness (“Correct step.”) was given and the sequence progressed to the next step.

Except for those in the NF condition, participants received Ver, Vib, or Ver+Vib feedback according to their assigned trial condition. Verbal feedback was given in the form of repeated verbal instructions. The vibrotactile stimulus was designed as a cyclic, on-off buzz with a cycle duration of 0.6 s, repeated four times (for a total duration of 2.4 s). No more than two feedback events were generated within a step. If the pose was still not correct after two feedback events, the step counter was reset to 1, and participants were asked to restart the pose from movement step 1 (*i*.*j*–1) with a verbal notification: “Let's start the pose over.” If the pose was still incorrect after four total feedback events, it was considered a *terminal failure*, and the pose was terminated with a verbal notification: “Let's move to a new pose.” At any point in time, participants could also choose to terminate a pose; poses terminated in this way were recorded as *withdrawals*.

#### 2.2.3. Pose difficulty levels

Selected yoga poses for the study were evaluated to determine their difficulty levels. Ten registered yoga teachers (7.7 ± 4.6 years of experience) were asked to review 48 yoga poses (three starting poses and 15 variation poses grouped under each starting pose; 15 × 3 = 45 poses) to assess the level of difficulty in performing each pose. The instructors were provided the name of the pose and pictures depicting the pose, and they were asked to rate each pose on a three-point scale: “Easy,” “Intermediate,” or “Difficult,” considering the extent of balance control and strength required to perform and hold the pose. They were asked to evaluate the difficulty level with reference to a novice practitioner who maintains good health status with no physical and visual impairment as the target performer. All participants provided written informed consent prior to participation, following the procedures approved by the university's Institutional Review Board.

Mean difficulty ratings across all instructors were calculated by converting the categorical levels into a numeric scale: “Easy” = 1, “Intermediate” = 2, and “Difficult” = 3. The 15 variant poses grouped under each starting pose were clustered into three categories based on the mean ratings, and thresholds of 1.30 and 2.20 were used to separate the three difficulty levels across all starting poses (Easy: 1.0–1.30, Intermediate: 1.31–2.20, Difficult: 2.21–3.0). Based on this clustering, there were 10, 22, and 16 poses in the easy, intermediate, and difficult categories, respectively, across all starting poses. The difficulty level of each pose is color-coded as green, yellow, or red, representing easy, intermediate, and difficult, respectively, in Supplementary Figures 1–6.

#### 2.2.4. Yoga learning trials

We created a total of 31 short yoga pose sequences, each composed of 4–6 poses of varying difficulty and based on a single starting pose. The pose difficulty levels were balanced within each sequence, so each sequence was composed of approximately 1–4 easy poses (median: 2), 0–4 intermediate poses (median: 2), and 0–4 difficult poses (median: 1). All sequences had at least one easy pose—at least the starting pose was easy—and at least one either intermediate or difficult pose, with a majority of sequences (61.3%) including all three difficulty levels. Each participant was assigned four randomly selected yoga sequences per starting pose, and a random permutation of the four feedback conditions was assigned to each set of four sequences. Thus, each participant experienced 12 short yoga sequences as their learning trials (3 starting poses × 4 feedback conditions = 12 sequences) in the study. Each trial took 3.0 ± 1.1 min to complete on average; the completion time varied with each individual's learning progress. Participants were given breaks of at least 2–3 min between trials. Pose sequences and names used for each trial are summarized in Supplementary Figures and organized by starting pose (Supplementary Figures 1–3).

### 2.3. Instrumentation

**Figure 3:**
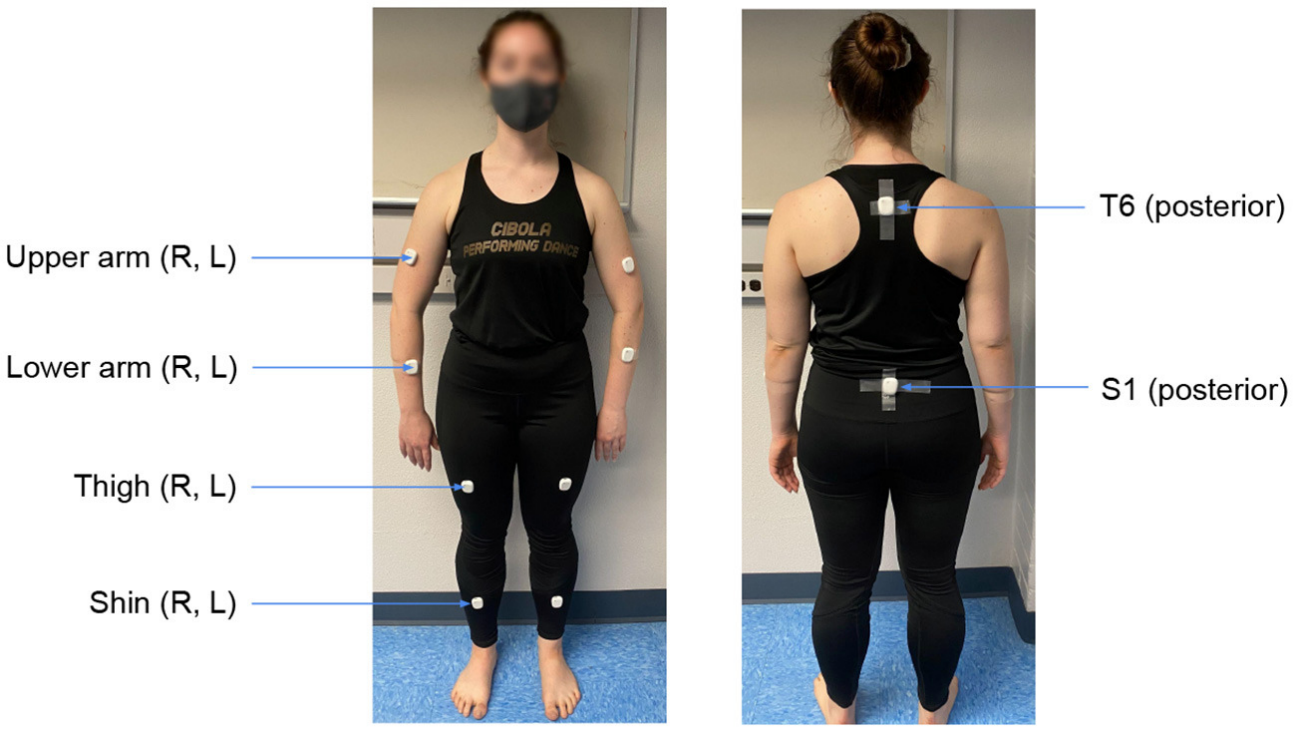
Ten anatomical locations used for sensor attachment. Sensors are attached on clothes just for demonstration purposes.

Participants were instrumented with ten commercial wearable inertial sensors combined with a micro coin vibration motor (MetaMotionR+, Mbientlab Inc., San Francisco, CA) during the experiment. The coin vibration motors (8 mm in diameter, operating voltage of 2.7–3.3 VDC) within the wearable sensors were used to send vibrotactile feedback to the participants. The vibrotactile stimulus was designed as a cyclic on-off feedback pattern with a cycle time of 0.6 s (0.3 s on followed by 0.3 s off; total time = 2.4 s) and a frequency of 165 Hz. A wearable sensor was attached over the sixth thoracic vertebra (T6), and another was attached over the first sacral vertebra (S1). Eight more sensors were attached bilaterally around the upper extremities (upper and lower arms) and the lower extremities (thighs and shins) as depicted in Figure 3. All 10 sensors were attached directly to the skin using hypoallergenic double-sided tape. The location of body parts for sensor attachment were selected based on a prior study conducted by the research team (50).

### 2.4. Performance measures

Three performance measures were obtained as dependent variables in this study; namely, pose completion level, feedback frequency, and time for pose correction.

**Pose completion level:** Pose completion was recorded at four levels: (1) correct, (2) correct after feedback, (3) terminal failure, and (4) withdrawal. If a participant completed a pose correctly without needing feedback at any movement step, the pose was recorded as “correct.” If a participant received at least one feedback event during any movement step and the feedback corrected the movement step(s), the pose was recorded as “correct after feedback.” If feedback events were unsuccessful in correcting a movement step after four attempts, this was recorded as a “terminal failure.” Lastly, if a participant voluntarily withdrew from a pose for any reason, including loss of balance, lack of flexibility, or low confidence, the pose was recorded as a “withdrawal.”

**Feedback frequency:** The number of feedback events provided before the successful completion of a pose was recorded as the feedback frequency. This data was recorded only for poses in the “correct after feedback” category.

**Figure 4:**
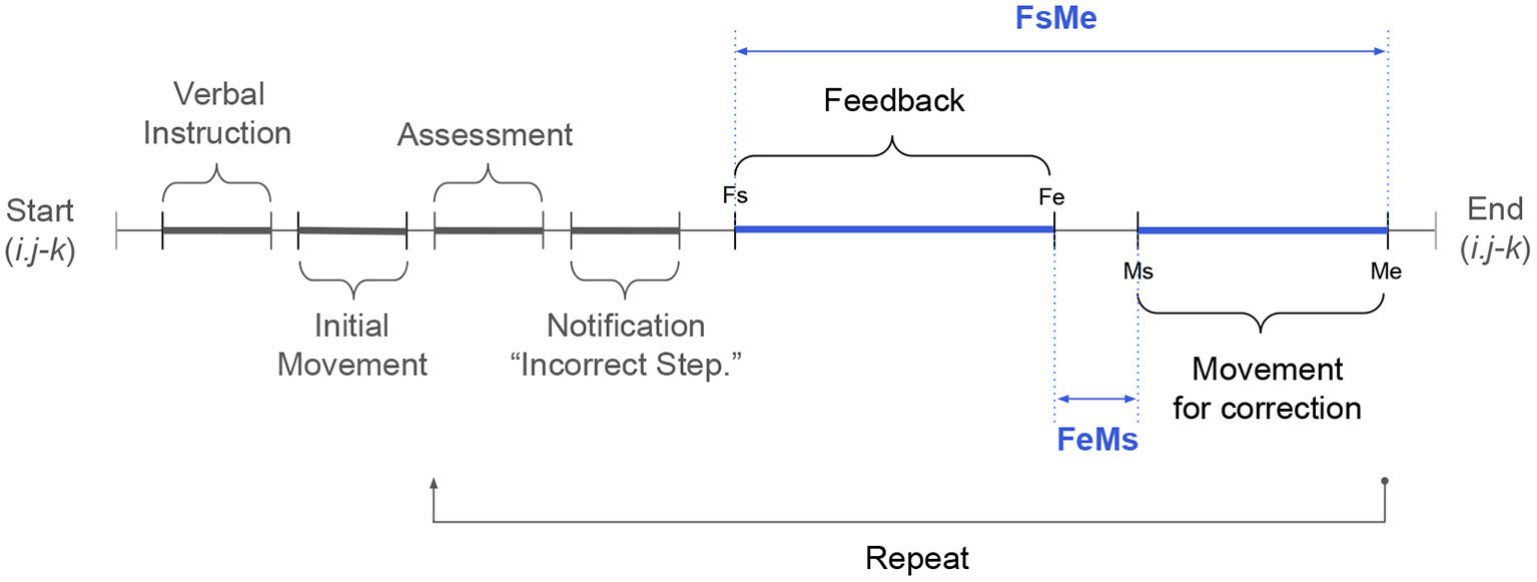
A timeline showing the sequence of events in the feedback system described in Figure 1.

**Time for pose correction:** Two time measures—FsMe and FeMs—were calculated to investigate the efficiency of feedback (Figure 4). FsMe indicates the time elapsed between the start of a feedback event (Fs) and the end of the associated corrective movement (Me). FsMe characterizes the time it takes for participants to finish correcting a pose from the start of the feedback. Thus, FsMe values serve as a means of comparing the time-efficiency of feedback events. FeMs refers to the time elapsed between the end of a feedback event (Fe) and the start of the associated corrective movement (Ms). We recorded FeMs to understand whether participants needed to perceive feedback events for their full duration before beginning to execute corrective movements. Thus, FeMs can be negative if a participant started moving their body while feedback was still being provided. Time measures were calculated only for poses in the “correct after feedback” category.

### 2.5. User preference ratings

After completing all of the yoga trials, participants ranked the feedback modalities (NF, Ver, Vib, and Ver+Vib) according to overall preference, with one being the most preferred and four being the least preferred. Participants were also asked to share their experience with the feedback system in an open-ended questionnaire.

### 2.6. Frequently corrected body segments

To explore the optimal sensor attachment locations, we counted the number of body segments that were frequently corrected based on the feedback provided throughout all yoga trials. From a practical perspective, limiting the number of sensors is a critical aspect of wearability when designing a wearable, sensor-based system (61). In our preliminary study, the important sensor attachment locations were investigated by identifying the location of body segments which perform critical movements in each yoga pose relative to the associated starting pose (50). The lower arms (R/L), upper arms (R/L), and right thigh were identified as the most critical body sites (50). The results from this study will be compared to discuss practical guidance toward the future development of wearable sensor-based exercise feedback systems.

### 2.7. Balance recovery and loss

Balance recovery and complete balance loss were recorded as separate events based on the video analysis. Balance recovery was an event in which a participant lost their balance or became unstable while performing a pose, but subsequently regained and maintained stability without falling or withdrawing from the pose. Balance loss, on the other hand, was an event in which a participant could not maintain or regain their stability during a pose, leading to withdrawal from the pose. For safety reasons, participants were encouraged to withdraw from a pose if maintaining balance became challenging at any time during the learning trials.

### 2.8. Statistical data analysis

Statistical analysis was performed using version 4.1.1 of the R programming language (62). First, descriptive summary statistics including mean and standard deviation (S.D.) were calculated for participant demographics (i.e., age, body mass, stature, and ABC score). For NVI participants, differences in demographic data between genders were tested using one-way ANOVAs (63) and non-parametric Kruskal-Wallis *H*-tests (64), as appropriate. Stature (*W* = 0.96, *p* = 0.51), body mass (*W* = 0.96, *p* = 0.59), and BMI (*W* = 0.96, *p* = 0.41) were normally distributed according to Shapiro-Wilk tests (65), so one-way ANOVAs were used for these variables. Meanwhile, age (*W* = 0.69, *p* < 0.05) and ABC score (*W* = 0.85, *p* < 0.05) were not normally distributed, so nonparametric Kruskal–Wallis *H*-tests (64) were performed. We chose to use the conventional significance level of α = 0.05 for these tests.

We tested our first hypothesis (*H*1: effectiveness) by testing for relationships between the following factors: (1) pose completion level and feedback type across and within each pose difficulty, and (2) feedback frequency and feedback type or difficulty level. To that end, we used nonparametric chi-squared tests of independence (66) as implemented by the R package *stats v3.6.2*. Significant differences between pose completion level and feedback type were examined further using multiple pairwise comparisons with the Bonferroni correction (67).

We tested our second hypothesis (*H*2: time-efficiency) with two-way ANOVAs (68) for each of FsMe and FeMs. Feedback type and pose difficulty level were the independent variables in both tests. Statistically significant effects of feedback and pose difficulty (*p* < 0.05) detected by the two-way ANOVAs were examined further using pairwise comparisons with Tukey's HSD *post-hoc* test (69). Tukey's HSD-adjusted α = 0.05 was used as the significance level.

Lastly, we tested our third hypothesis (*H*3: user preference ratings) with a non-parametric Kruskal-Wallis test (70) as implemented by the R package *Rcompanion v2.3.7* (71). Rank-order preferences were converted to an ordinal scale (e.g., least preferred: 1, most preferred: 4) for testing. Statistically significant results (*p* < 0.05) were examined further using pairwise comparisons with the *post-hoc* Dunn-Bonferroni test (72). The proportions of user preference ratings out of all respondents for each feedback type were also calculated.

## 3. Results

Demographic information for the NVI participants (*n* = 22) is summarized in Table 1. Stature was the only variable for which we observed a statistically significant difference between male and female participants: *F*_(1, 20)_ = 19.57, *p* < 0.001. Other demographic variables including age [χ^2^_(1, 22)_ = 0.02, *p* = 0.89], body mass [*F*_(1, 20)_ = 2.58, *p* = 0.12], BMI [*F*_(1, 20)_ = 0.07, *p* = 0.8], and ABC score [χ^2^_(1, 22)_ = 0.11, *p* = 0.71] did not differ significantly between genders, so further statistical analyses were performed by pooling the data for both genders.

**Table 1:** Participant demographics (mean ± S.D.; *n* = 22).

	**Male (*n* = 9)**	**Female (*n* = 13)**	**Total (*n* = 22)**
Age (years)	26.6 ± 10.1	28.4 ± 15.1	27.6 ± 13.0
Stature (mm)^*^	1797.8 ± 69.0^a^	1684.7 ± 51.1^b^	1731.0 ± 80.9
Body mass (kg)	78.7 ± 15.2	67.5 ± 16.8	72.1 ± 16.8
BMI (kg/m^2^)	24.4 ± 4.7	23.8 ± 5.7	24.0 ± 5.2
ABC (score: 0–100)	97.0 ± 3.0	96.9 ± 2.3	96.9 ± 2.5

* *p* < 0.05. Letter superscripts indicate significant pairwise differences at the α = 0.05 level.

The participants performed a combined total of 1,256 yoga poses (591 easy poses, 406 intermediate poses, and 259 difficult poses) and 3,777 movement steps. The following sections summarize the results on feedback effectiveness, time-efficiency, and user preference ratings for different types of feedback modalities and yoga pose difficulty levels.

### 3.1. Feedback effectiveness (H_1_)

#### 3.1.1. Pose completion level

**Figure 5:**
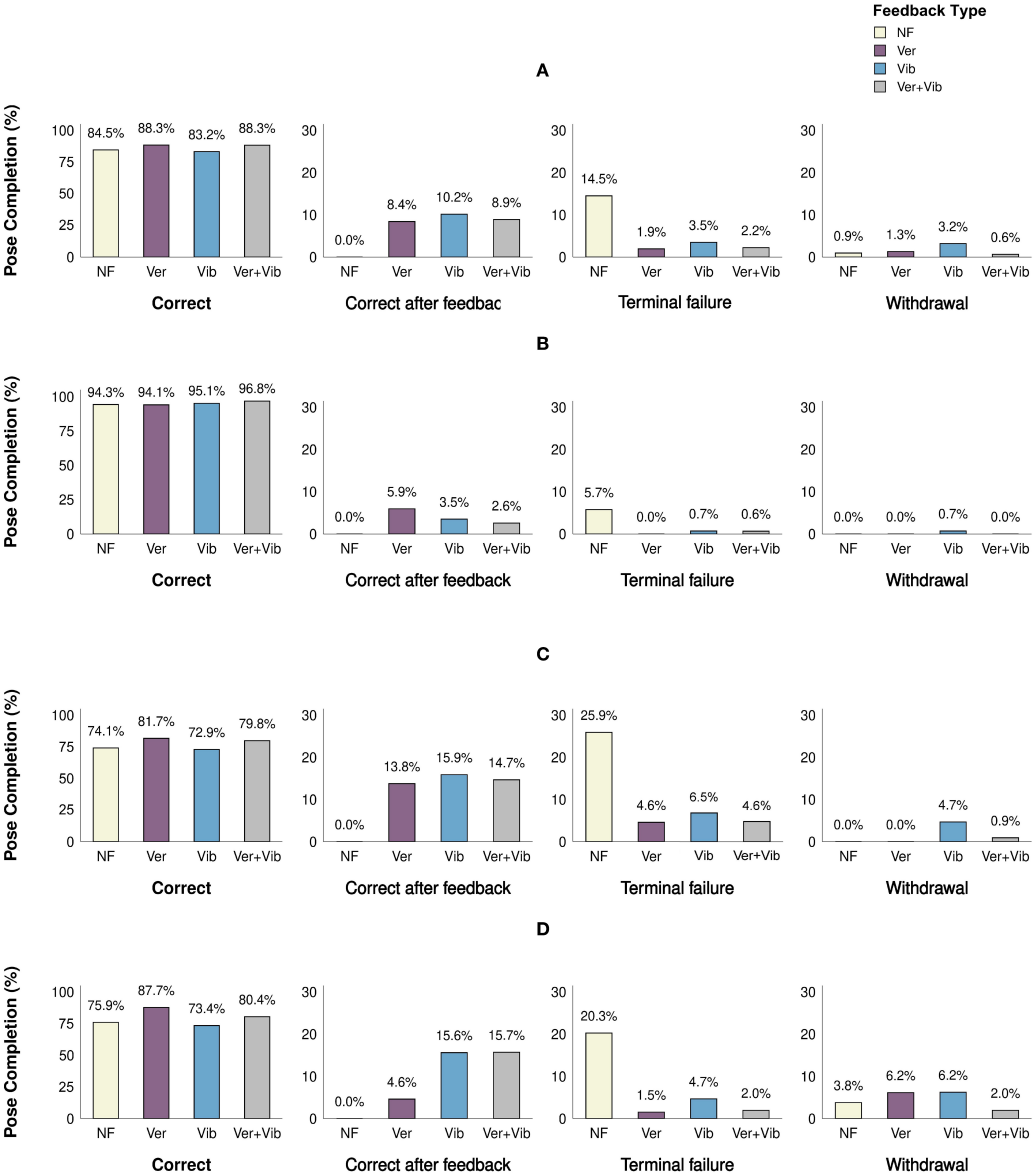
Pose completion level (%) for different feedback types. Letters indicate significant pairwise differences at *p* < 0.05 within each pose completion level category within the bar chart. **(A)** Combined, **(B)** easy, **(C)** intermediate, and **(D)** difficult.

The pose completion level (%) across all yoga poses performed in the study is shown for each of the four feedback types in Figure 5A, and then broken down by pose difficulty level in Figures 5B–D for easy, intermediate, and difficult poses, respectively. Pose completion level (%) is depicted separately for four categories: (1) correct, (2) correct after feedback, (3) terminal failure, and (4) withdrawal. Across all 1,256 poses, 1,081 poses (86.1%) were performed correctly on the first attempt, 86 poses (6.8%) were corrected after feedback, and 70 poses (5.6%) were terminally failed because they could not be corrected. Additionally, participants withdrew from 19 poses (1.5%). The feedback system was able to correct a total of 156 poses (86 + 70 poses; 12.4%); the rest of the poses were either performed correctly on the first try or withdrawn from. Given that participants received no feedback in the NF condition, with the exception of the 46 poses that were terminally failed in the NF condition, a total of 110 poses (8.8%) were candidates for correction by the feedback system. These results show that compared with the NF condition, feedback corrected an additional 11.2% of poses (NF: 84.5% vs. Feedback: 95.7%).

Across all difficulty levels (Figure 5A), there was no significant difference in the frequency of correct poses [χ^2^ (3, *N* = 1,081) = 0.52, *p* = 0.91], correct poses after feedback [χ^2^ (2, *N* = 86) = 0.65, *p* = 0.74], and withdrawals [χ^2^ (3, *N* = 19) = 8.16, *p* = 0.05] among feedback types. Only the proportion of terminal failures differed significantly across difficulty levels [χ^2^ (3, *N* = 70) = 62.69, *p* < 0.001]. The *post-hoc* test identified a significantly higher proportion of terminal failures in the NF condition compared to all feedback conditions, suggesting that feedback was effective in correcting incorrectly performed poses, thereby reducing terminal failures. No significant difference in the proportion of terminal failures was observed among different types of feedback (Ver, Vib, Ver+Vib).

Similar trends were observed within difficulty levels (Figures 5B–D). Within each difficulty level, the rate of terminal failure was found to differ significantly among feedback types [Easy: χ^2^ (2, *N* = 11) = 11.64, *p* = 0.005, Intermediate: χ^2^ (3, *N* = 38) = 18.84, *p* < 0.001, Difficult: χ^2^ (3, *N* = 21) = 29.86, *p* < 0.001]. Pairwise *post-hoc* tests identified a difference between NF and all other feedback conditions in each case, with the notable exception that there were no terminal failures at all in the Ver condition for easy poses. In summary, feedback was effective in correcting yoga poses compared to no feedback, regardless of pose difficulty level and feedback type. However, there was no significant difference in effectiveness among different feedback modalities.

#### 3.1.2. Frequency of feedback

**Figure 6:**
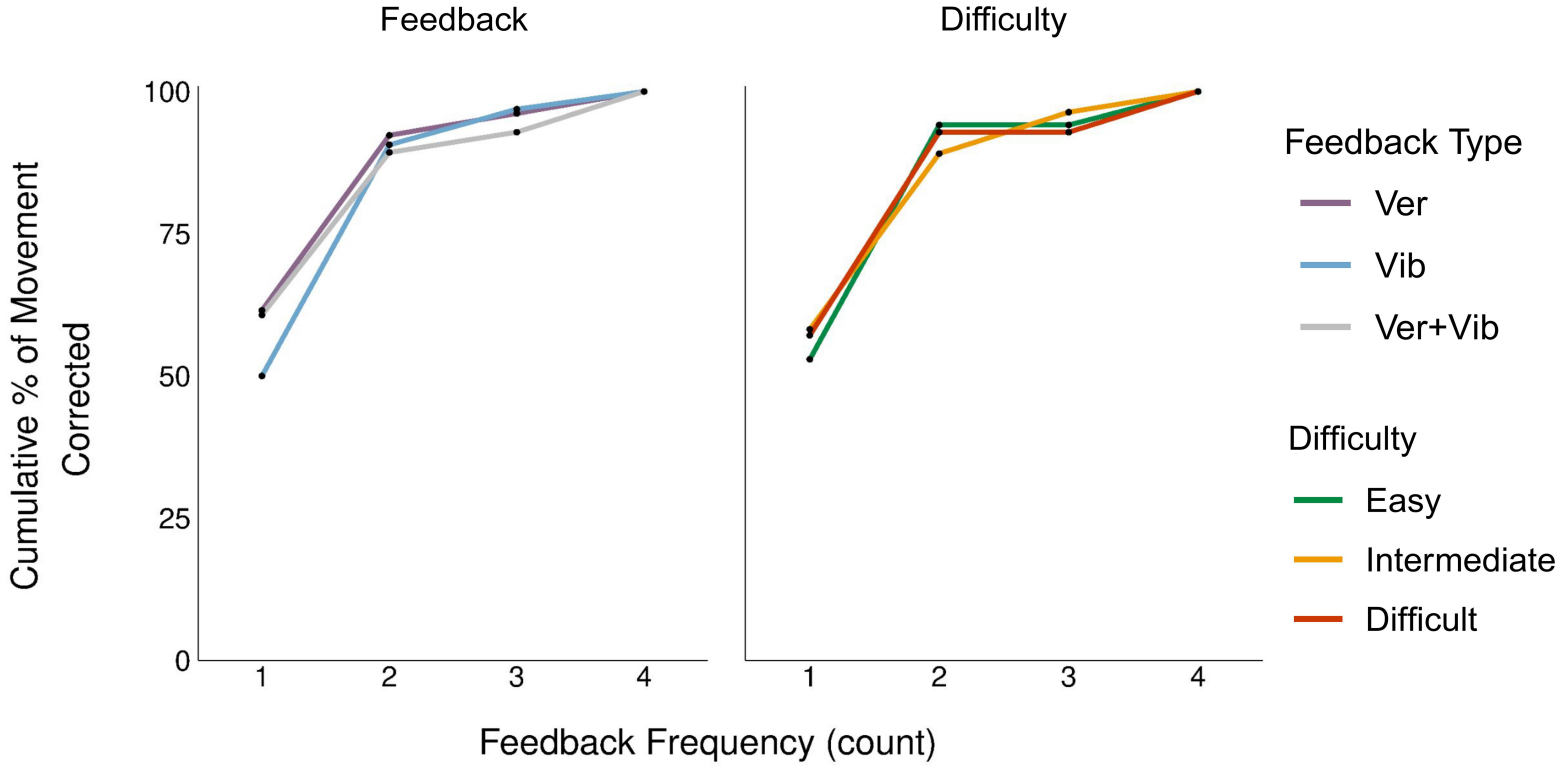
Frequency of feedback events required for pose correction (cumulative % of movement corrected).

Figure 6 shows the cumulative percentages of corrected movement steps across feedback modalities for each feedback frequency level (i.e., event count) from 1 to 4. For example, a total of 32 movement steps were corrected with vibrotactile feedback. Among those 32 steps, 16 were corrected with one feedback event (50.0%), 13 with two feedback events (40.6%), 2 with three feedback events (6.3%), and 1 with four feedback events (3.1%). Given that the total number of steps corrected varied across feedback types and pose difficulty levels, we calculated the proportion of steps corrected for each feedback frequency level. The graph to the left (Figure 6) is broken down by feedback type, while the one on the right (Figure 6) is broken down by pose difficulty. The mean ± SD frequency counts for Ver, Vib, and Ver+Vib feedback were 1.5 ± 0.8, 1.6 ± 0.8, and 1.6 ± 0.9, respectively. The mean ± SD frequency counts for Easy, Intermediate and Difficult poses were 1.6 ± 0.8, 1.6 ± 0.8, and 1.4 ± 0.7, respectively. Chi-squared tests showed no statistically significant difference among feedback types [χ^2^ (6, *N* = 86) = 2.01, *p* = 0.92] or pose difficulty levels [χ^2^ (6, *N* = 86) = 4.16, *p* = 0.66]. The results show that most corrections required only one or two feedback events, and all feedback types showed similar effectiveness.

### 3.2. Time-efficiency (H_2_)

**Figure 7:**
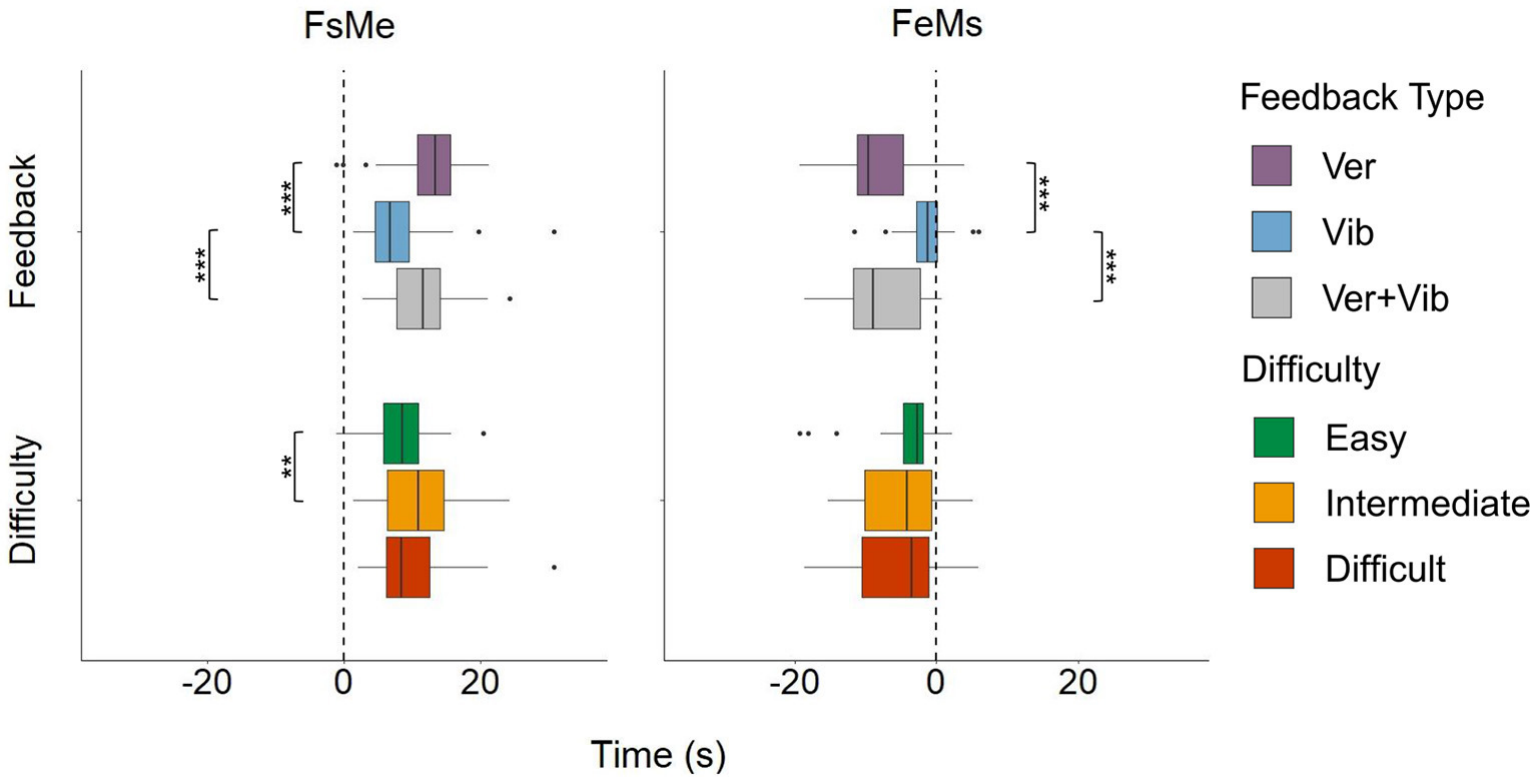
FsMe and FeMs by feedback type and pose difficulty level. “**” and “***” indicates statistically significant pairwise comparisons for which *p* < 0.01 and *p* < 0.001, respectively.

A single vibrotactile feedback event had a duration of 2.4 s (Fs to Fe). However, there were system delays between sending multiple vibrations to multiple body sites; thus, the duration varied (3.6 ± 1.4 s) in the Vib condition. For the Ver and Ver+Vib conditions, the feedback duration depended on the length of the verbal instruction, with an average duration of 11.4 ± 3.6 s (range: 2.9–19.5 s). A one-way ANOVA showed that the length of the feedback (Fs to Fe) varied significantly among feedback types [*F*_(2, 227)_ = 202.2, *p* < 0.001]. On average, Vib was 7.9 s shorter than Ver (95% CI: [−9.0, −6.7], adj. *p* < 0.001) and 7.7 s shorter than Ver+Vib (95% CI: [−8.7, −6.6], adj. *p* < 0.001). Figure 7 summarizes the values of two time measures (FsMe and FeMs) by feedback type and pose difficulty level, separately. Average time measures across all poses were 10.0 ± 5.0 s for FsMe and −5.1 ± 5.3 s for FeMs.

Two-way ANOVAs showed statistically significant differences in FsMe among feedback types [*F*_(2, 227)_ = 34.5, *p* < 0.001] and pose difficulty levels [*F*_(2, 227)_ = 5.2, *p* = 0.006]. Within feedback types, the Vib condition showed the fastest response (FsMe in Vib: 7.6 ± 4.3 s) compared to Ver (12.4 ± 4.8 s) by 4.7 s (95% CI: [−6.2, −3.2], *adj*.*p* < 0.001), and Ver+Vib (11.3 ± 4.6 s) by 3.7 s (95% CI: [−5.1, −2.2], adj. *p* < 0.001). On average, this is a reduction of response time from the start of a feedback event to the end of the pose correction by 38.2% and 32.7% from the Ver and Ver+Vib conditions, respectively. Within pose difficulty levels, participants responded more quickly when performing easy poses (8.4 ± 4.2 s) compared to intermediate poses (10.6 ± 4.9 s), with an average difference of 2.2 s (95% CI: [−4.0, −0.5], adj. *p* = 0.007). We observed no other significant differences between pairs.

Feedback type significantly affected FeMs [*F*_(2, 227)_ = 88.2, *p* < 0.001], while FeMs did not vary significantly by pose difficulty level [*F*_(2, 227)_ = 1.5, *p* = 0.2]. Unlike FsMe, negative times were observed in FeMs, indicating that participants started correcting their poses before feedback events had ended. Ver feedback showed the largest negative time (−8.1 ± 4.9 s), which was significantly lower than Vib (−1.5 ± 2.6 s) by 6.7 s (95% CI: [−8.1, −5.3], adj. *p* < 0.001). Ver+Vib also showed a significantly larger negative time (−7.6 ± 5.4 s) compared to Vib, with a difference of 6.2 s (95% CI: [−7.5, −4.8], adj. *p* < 0.001). Considering that the feedback duration differed across feedback types, differences in FsMe between Vib and Ver (or Ver+Vib) can be interpreted as consequences of the feedback duration difference (Ver and Ver+Vib are 7.8 s longer than Vib, on average). Overall, a larger negative FeMs in Ver and Ver+Vib compared to Vib indicates that there were some anticipatory movements—that is, the participants corrected movement steps before the feedback ended—in the Ver feedback, due mainly to the length of the feedback. Overall, Vib feedback was the most time-efficient option out of all feedback modalities because it was the shortest in time (Fs to Fe), produced the fastest pose corrections (FsMe), and exhibited the fewest anticipatory movements (FeMs).

### 3.3. User preference (*H*3)

**Figure 8:**
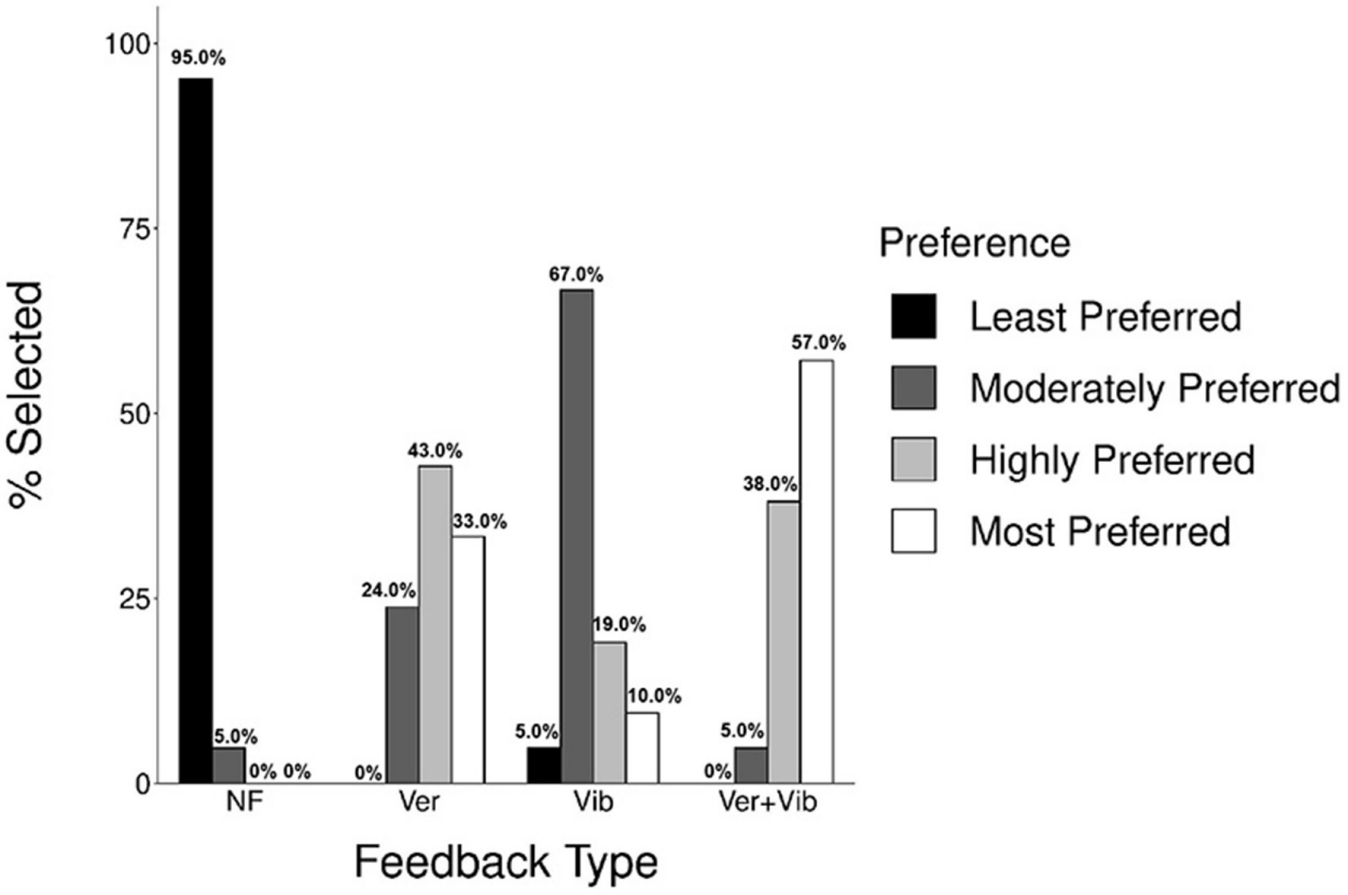
Participants' preferences for different feedback types (*n* = 21).

A summary of the participants' rank-order preferences for different types of feedback is shown in Figure 8. A majority of the participants (95%) identified the NF condition as the least preferred option, while multimodal (Ver+Vib) feedback was most strongly preferred (57.0%), followed by Ver (33.0%). A Kruskal-Wallis test showed a significant difference in the rank-order of participants' preferences among feedback types; χ^2^ (3, *N* = 84) = 61.47, *p* < 0.001. All pairwise *post-hoc* comparisons showed significant differences in rank-order preference among different feedback types with the exception of the comparison between Ver and Ver+Vib feedback (adj. *p* = 1.0). Thus, Ver and multimodal (Ver+Vib) feedback were most strongly preferred over Vib feedback, and NF was the least preferred option (i.e., Ver > Ver+Vib > Vib > NF).

In an open-ended questionnaire asking about participants' overall experience with the feedback system, some reported that Vib feedback was particularly helpful in understanding which specific body parts needed correction. However, since the information was limited to simply alerting participants to the incorrect body location with a single tactor, participants found the information challenging to interpret (i.e., they understood what was wrong, but not how to fix it) compared to Ver or multimodal (Ver+Vib) feedback.

### 3.4. Body segments with frequent correction

**Figure 9:**
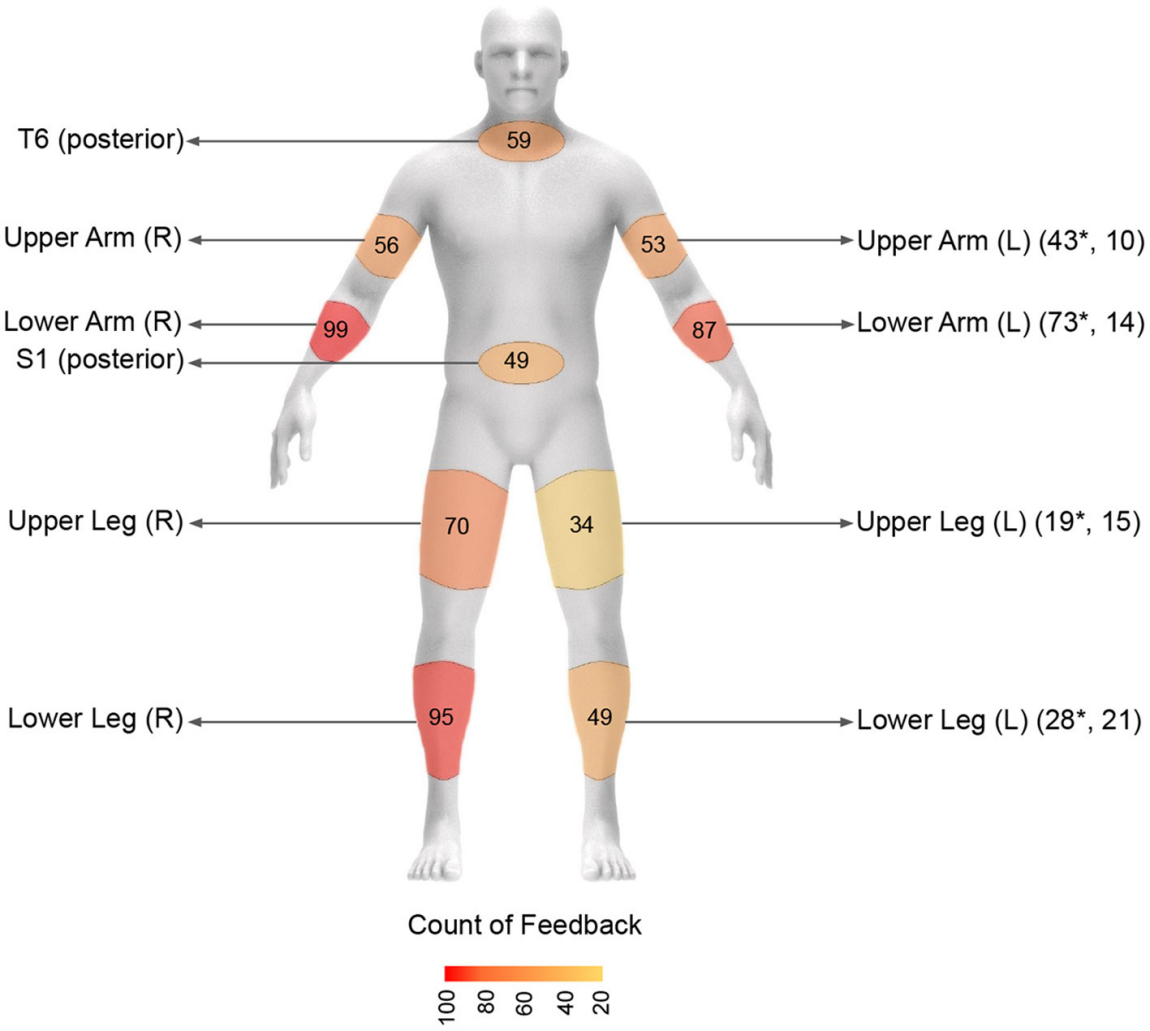
Total counts of feedback provided in the selected body segments across all feedback trials. Left side body segment is indicated by * if the left side posture is symmetric to the right side.

Figure 9 shows the total counts of feedback provided in different body segments where sensors were attached during the yoga trials. The lower arm (R/L) and lower leg of the right side of the body were the most frequently corrected body parts (99, 87, and 95 total counts, respectively), followed by the right upper leg (70 counts). On the other hand, the left upper and lower leg and lower back were the least frequently corrected body segments (34, 49, and 49 total counts, respectively).

## 4. Pilot study: Participants with vision impairment (VI)

Once the feasibility and safety of the feedback system was tested with NVI participants, we pilot-tested the system with our target user population: people with vision impairments (VI). A total of four VI participants (1 male and 3 females, age: 43 ± 8.7 years) were recruited to conduct a pilot test in the same manner discussed in Section 2. Participants were screened for their age (≥18 years), ability to walk and stand without pain and discomfort, and novice yogi status, in the same way described in Section 2.1 for NVI participants. The level of visual impairment was used as an additional inclusion criterion. Participants were included if they had a vision impairment ranging from moderate low vision to total blindness based on their self-reported visual acuity. Self-reported confidence in balance was obtained using the ABC scale (55) and all participants provided written informed consent prior to participation, which was read aloud by our researcher when requested.

Due to the limited volume of participant data collected from the VI group (as this study was performed in early 2021 when social distancing measures due to the global pandemic were in effect, limiting our access to outreach and recruitment of potential participants), our investigation is mostly focused on discussing the overall trend in descriptive summary statistics, including mean and standard deviation (S.D.), and qualitative analyses. No inferential statistical analysis was performed for the data collected from this group except for the pose completion level comparison and balance loss and recovery comparison between the NVI and VI participants. To compare pose completion levels, balance loss, and recovery between NVI and VI participants, nonparametric chi-squared tests of independence (66) were used, as the sample sizes between the groups [*n* = 22 (NVI), 4 (VI)] were unbalanced.

### 4.1. Results

Demographics for VI participants (*n* = 4; 1 male, 3 females) are summarized in Table 2. Two of the participants had total blindness, whereas the other two participants had near-total blindness and severe low vision. The four participants performed a combined total of 233 yoga poses (105 easy poses, 71 intermediate poses, and 57 difficult poses) and 796 movement steps.

**Table 2:** Summary demographics of VI participants (*n* = 4).

	**P1**	**P2**	**P3**	**P4**	**Total (mean ±S.D.)**
Gender	M	F	F	F	1 M, 3 F
Age (years)	30	48	47	47	43 ± 8.7
Stature (mm)	1,880	1,635	1,630	1,580	1681.3 ± 134.8
Body mass (kg)	119.7	71.6	62.8	54.4	77.1 ± 29.2
BMI (kg/m^2^)	33.9	26.8	23.6	21.8	26.5 ± 5.3
ABC (score: 0–100)	90	65.7	80	90.6	81.6 ± 11.6
Vision impairment level	< 20/1,000	Between 20/200 to 20/400	No light perception	No light perception	–
	(near-total blindness)	(severe low vision)	(total blindness)	(total blindness)
Time with impairment	1–3 years	More than 10 years	Since birth	Since birth	–

#### 4.1.1. Feedback effectiveness (*H*1)

**Figure 10:**
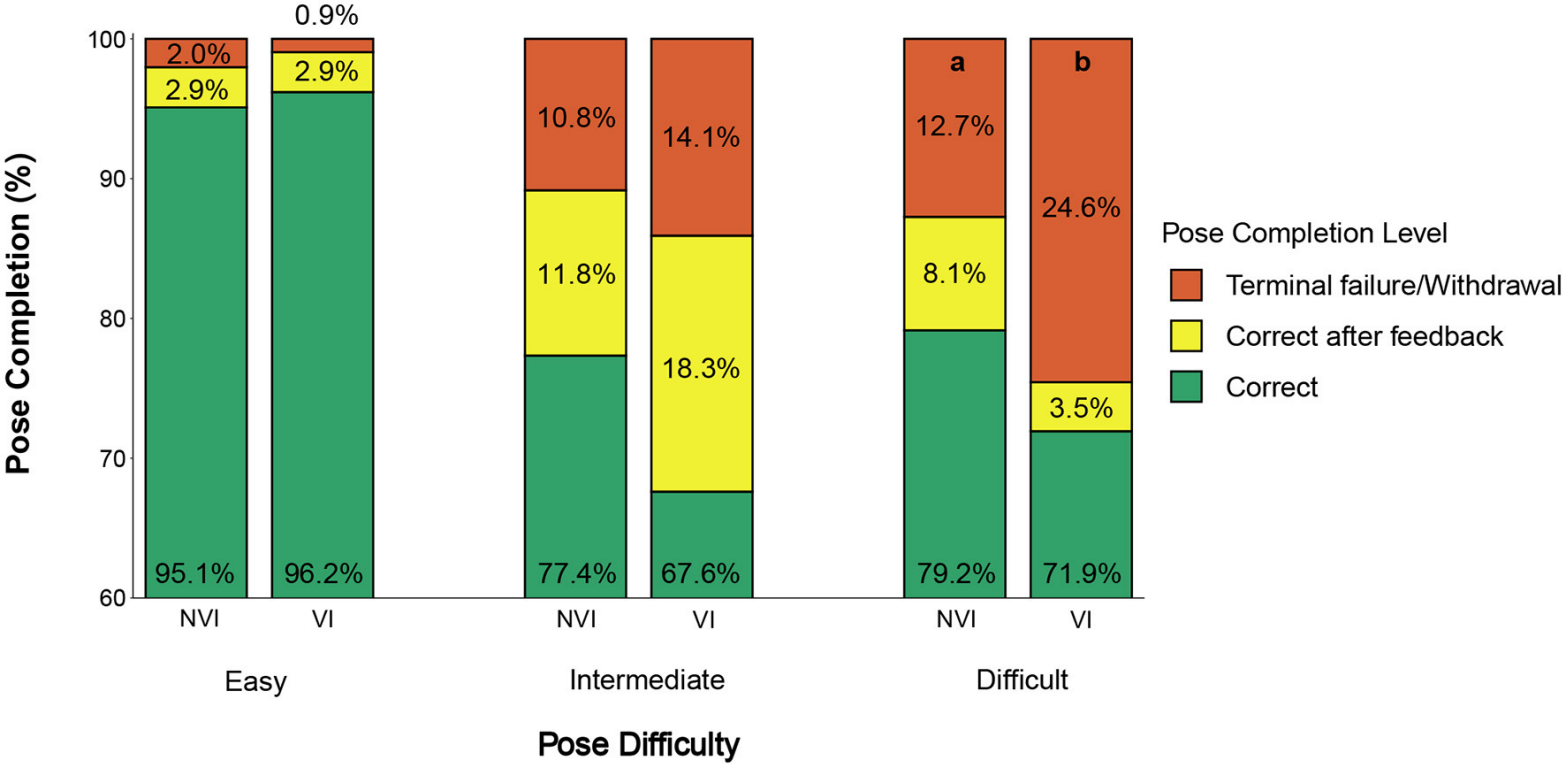
Comparison of pose completion rates (%) for NVI and VI participants. Letters indicate significant pairwise differences between NVI and VI within the same category at *p* < 0.05.

Figure 10 presents pose completion rates (%) between the NVI and VI groups across all yoga poses performed in the study for each of the easy, intermediate and difficult pose categories. The pose completion rate (%) for the comparison is shown for three categories; namely, (1) correct, (2) correct after feedback and (3) terminal failure/withdrawal. Out of the 233 poses (easy = 105, intermediate = 71, difficult = 57) that VI participants performed, 190 poses (81.5%) were performed correctly on the first attempt, 18 poses (7.7%) were corrected after feedback, 16 poses (6.9%) were terminal failures, and participants withdrew from 9 poses (3.9%) across all difficulty levels. Overall, feedback was able to correct 6.7% of initially incorrect poses for the VI participants, which is 4.5% lower than the NVI participants (11.2%). The VI participants also recorded 24.6% (14 out of 57 poses) terminal failures/withdrawals in the difficult poses which is 11.9% higher compared to the NVI participants who recorded 12.7% (33 out of 261 poses) terminal failures/withdrawals in the difficult poses [χ^2^ (1, *N* = 47) = 4.39, *p* = 0.04]. Other than the terminal failure/withdrawal rate for difficult poses, the pose completion rate within each category did not differ significantly between the NVI and VI participants.

#### 4.1.2. Time efficiency (*H*2)

Average ± S.D. times for FsMe and FeMs were 10.9 ± 4.8 and −5.95 ± 5.1 s, respectively. A similar pattern was observed with the VI participants in time measures compared to the NVI participants, where Vib feedback yielded the shortest FsMe and the shortest negative FeMs. Similarly, easy poses yielded the shortest FsMe and the shortest negative FeMs.

#### 4.1.3. User preference (*H*3)

All the participants ranked NF as the least preferred option. Two participants considered the multimodal feedback as the most preferred, while the other two participants most preferred verbal-only feedback.

#### 4.1.4. Balance loss and recovery

**Figure 11:**
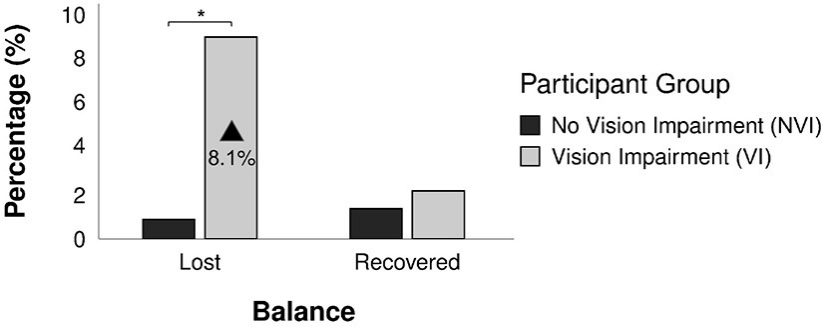
Comparison of balance loss and recovery (%) of NVI and VI participants. *Indicates statistically significant pairwise comparisons for which *p* < 0.05.

Figure 11 shows a comparison of balance loss and recovery rates between the NVI and VI participants. NVI participants lost their balance during 11 poses (0.9%) and recovered their balance during 17 (1.4%) poses out of the combined 1,256 poses they performed. VI participants lost their balance during 21 poses (9.0%) and recovered their balance during 5 poses (2.1%) out of the combined 233 poses they performed. The comparison showed a significantly higher occurrence of balance loss during the trials with VI participants [χ^2^ (1, *N* = 32) = 60.55, *p* < 0.001], while no significant difference was observed for balance recovery between the participant groups [χ^2^ (1, *N* = 22) = 0.83, *p* = 0.36].

## 5. Discussion

The objective of this study was to assess the effectiveness of vibrotactile and multimodal feedback in guiding a remote yoga practice. The study results supported our hypotheses (*H*1: feedback effectiveness, *H*2: time-efficiency, and *H*3: user preference); namely, that either vibrotactile or multimodal (Ver+Vib) feedback would be more effective than no feedback, vibrotactile feedback would be more time-efficient than verbal feedback, and users would prefer any type of feedback over no feedback at all. All types of feedback were found to be effective in correcting movements compared to no feedback, while pose correction with vibrotactile feedback was significantly more time-efficient than verbal and multimodal (Ver+Vib) feedback. Participants most preferred multimodal feedback (Ver+Vib) or verbal feedback over vibrotactile feedback, with no feedback at all as the least preferred option. Although statistical comparisons were performed with limitations between NVI and VI participants due to the sample size disparity, the observed trends were similar for the performance measures. The following sections discuss the results of testing the study hypotheses and their implications as guidance for future training platform development with vibrotactile or multimodal feedback.

### 5.1. Nonvisual feedback on yoga practice

#### 5.1.1. Feedback effectiveness (*H*1)

Personalized non-visual feedback in response to a participant's performance was effective in guiding a remote yoga practice. This hypothesis was supported by a decrease in the “terminal failure/withdrawal” category for all feedback types compared to no feedback; this is a consequence of the increase in corrected poses after feedback, regardless of pose difficulty level. The percentage of corrected poses after feedback was higher in intermediate (11.8 and 18.3% for the NVI and VI groups, respectively) and difficult poses (8.1 and 3.5% for the NVI and VI groups, respectively) compared to easy poses (2.9 and 2.9% for the NVI and VI groups, respectively), suggesting that feedback is helpful with more challenging poses, as those poses are more likely to require correction of the initial attempt. Prior studies support our findings in which providing quantitative and personalized feedback in response to the movement of a user improves performance (73). In a remote learning setup, activities like aerobic exercise (16, 74), balance and strength training (75), rowing (76), and tennis (77) all showed positive performance results when participants received personalized performance feedback. For example, both vibrotactile and visual feedback significantly improved motion accuracy and reduced the average number of errors in a remote balance training activity compared to no feedback (78). Other similar application areas, such as physical rehabilitation (79, 80), also showed positive performance results with personalized sensory feedback. Although there are not many comparison studies performed in an area of physical training using vibrotactile or multimodal feedback with people with VI, personalized auditory feedback in an exergame for this population showed improved performance in practicing yoga poses (60). Participants also required fewer correctional feedback events over time, indicating effective learning with such personalized feedback (60).

Feedback frequency did not differ significantly across feedback types. This result indicates that all feedback types were comparably effective in correcting movements, and there was no one feedback type that was more effective than another. A majority of the steps (78 out of 86, 90.7%) were corrected within two feedback events with the NVI participants. The average counts of feedback events (1.5, 1.6, and 1.6, for Ver, Vib, and Ver+Vib, respectively) suggest that more than one feedback event was needed on average. This could indicate potential ambiguity in the provided feedback, which might have confused participants as to how to fix a given pose. The results also suggest that additional feedback events beyond the second may not offer any additional benefit; overall, third and fourth events corrected only a minimal number of incorrect movements (4.7% additional corrections for both three and four repetitions).

#### 5.1.2. Time efficiency (*H*2)

Vibrotactile was the most time-efficient feedback type among the three tested, as the feedback duration was the shortest and it produced the fastest pose corrections while also yielding the fewest anticipatory movements. Time-efficiency in feedback perception is important in applications that require time-sensitive responses. Physical training platforms are one such application, as a potential delay in perceiving and reacting to feedback can elevate safety concerns. For example, muscle fatigue and balance loss can develop while participants are performing an exercise task, and delays in perceiving feedback could increase potential injuries and fall risks, especially among older adults and people with sensory impairments. In the study, VI participants lost balance during 9.0% of the total poses (regardless of the feedback condition), which was 8.1% higher than the NVI participants. This result is aligned with prior studies that reported higher levels of postural instability (81) and increased center-of-gravity velocity (82) among people with restricted vision. The ABC score also showed different levels of self-reported confidence in balance among NVI (96.9) vs. VI (81.6) participant groups in our study, which might have contributed to the greater incidence of balance loss during the trials.

In the yoga activity we studied in this paper, participants were often required to maintain postural balance in one-legged standing postures [e.g., Tree (Pose 1.1), Clasped Eagle (Pose 1.2), Standing One Leg Lifted (Pose 1.3), and Dancer (Pose 1.4) in Supplementary Figure 1], and a protracted delay in perceiving and reacting to feedback in such a pose may increase discomfort and injury risks, including falls. Furthermore, participants may lose focus and interest if they experience long and repetitive feedback events, which could make the remote exercise experience boring and monotonous. In an arm movement training system, vibrotactile feedback was found to be efficient in accelerating motion learning (by 23%) and increasing response accuracy (by 27%) compared with visual feedback, which supports our study's findings (31). To provide safer training environments for remote exercise among the population with VI, the use of a fast and efficient feedback modality must be emphasized. In summary, the time-efficient nature of vibrotactile feedback can help with improving remote physical training while reducing potential adverse effects.

#### 5.1.3. User preference (*H*3)

Trends of preferring multimodal (verbal and vibrotactile) feedback over unimodal feedback were observed in prior studies. A combination of multisensory cues has been found to improve task performance, accuracy, and spatial attention (35, 83, 84). Providing clear and easily understandable movement guidance through vibrotactile arrays and movement sonification can also improve user satisfaction. For specific populations experiencing motor and cognitive function decline (e.g., older adults with age-related degeneration), multimodal feedback improved learning performance and was more strongly preferred (vs. unimodal) during physical tasks (85).

NVI participants strongly preferred multimodal feedback over verbal-only feedback, while VI participants were equally split between multimodal and verbal-only feedback. In previous studies, people with VI preferred multimodal feedback over unimodal while performing physical exercise [e.g., tennis exergames; (15)]. Since people with VI rely heavily on spatial hearing in daily activities, such as navigation (86), they tend to prefer any type of feedback system that comes with auditory guidance, whether it is unimodal or multimodal (e.g., augmented with vibrotactile feedback).

### 5.2. Potential safety issues with remote yoga practice

Pose completion rates between participant groups did not differ significantly for the poses with easy and intermediate difficulty levels. However, VI participants showed significantly higher terminal failure and withdrawal rates (11.9% higher than NVI participants) while performing difficult poses. As some of the difficult poses require participants to maintain balance in one-legged posture or require considerable core body strength for maintenance, VI participants either lost their balance or withdrew from the poses because they could not hold them. In the pilot study with VI participants, participants were allowed to lean against the wall or hold onto a chair placed nearby as a safety precaution. In practice, remote exercise platforms targeting VI participants will need to consider customization and filtering of poses based on participants' balance capabilities and suggestions for props and assistive features (e.g., walls, handles, chairs) to prevent falls and balance loss during the practice. Scores from a balance measuring scale [e.g., sensory organization test (87), Berg balance scale (88), activity-specific balance confidence scale (55)] can be used to assess baseline balance capabilities and incorporated in the system to filter the available yoga poses, making the remote exercise session more engaging and safe considering the higher incidence of balance loss observed among VI participants in this study.

### 5.3. Sensor attachment location

Limiting the number of sensors for remote exercise is a practically important issue. The location of sensor attachment is also crucial for wearability, as users may find it difficult to attach sensors to certain body segments [e.g., posterior upper back (T6)] on their own. Among the eight sensor attachment locations tested in this study, lower arm (R), lower leg (R), and lower arm (L) were found to be the most corrected body segments. This finding is similar to our preliminary study performed with 17 yoga poses [compared to 48 poses in this study; (50)]. A future study may consider using wristbands or ankle bands with easily attachable straps for sensor attachment and vibrotactile feedback by embedding the IMU and coin motor units within the bands to improve wearability.

In addition, the corrected body segments on the left/nondominant side of the body was often symmetric to the right/dominant side of the body as indicated in Figure 9. This information can be used to potentially remove any sensors on the left/non-dominant side and instead provide verbal feedback that indicates whether the correction needs to be made unilaterally or bilaterally along with the vibrotactile feedback. Overall, there is a need to reduce the number of sensors for practicality, usability, and wearability (61), and our investigation suggests lower arm (R) and lower leg (R) as the most crucial body segments that need feedback; the lower arm (L) can be mirrored with the lower arm (R).

### 5.4. Additional future design considerations

In this study, our investigation was specific to the effectiveness of feedback in pose correction, but further studies are needed to investigate whether such feedback also positively affects participants' motivation and program adherence in exercise training (89). Though existing remote exercise platforms provide an accessible solution for people with VI for exercise participation, these platforms currently do not provide adequate personalized performance assessments and feedback to individual participants during an exercise session. Technological advancements and the reduced cost of markerless motion capture systems [e.g., Microsoft Kinect; (26)], computer vision techniques [e.g., Openpose; (90)], and wearable sensing technologies (91) have created opportunities for improvement in assessing individual performance and providing real-time feedback for effective remote training using these learning modalities. Exercise customization and adaptation through adequate assessment and feedback are especially important in accommodating users with different physical capabilities [e.g., experience, health condition, strength; (92)] and increasing levels of motivation and satisfaction, which promotes long-term adherence (93).

Since multimodal feedback combining vibrotactile feedback with another sensory modality provides a better learning experience than unimodal feedback (76), creating more time-efficient multimodal feedback (e.g., Ver+Vib) might be a solution to enhance both the effectiveness (*H*1) and the overall time-efficiency (*H*2) of remote learning. In this study, verbal feedback was lengthy, as it took the form of a repeated full, step-by-step instruction. Alternative forms of verbal feedback could address this issue. For example, simply repeating the body part and indicating the direction of movement (e.g., “Right arm, up”) would significantly reduce the duration of the verbal feedback. Movement sonification is another way to provide time-efficient auditory feedback. Movement sonification maps movement variables to a parameter of sound (e.g., pitch or volume), which can help to enhance motor learning activities and performance (94–97). In prior studies, the use of sonification optimized movement control and execution in complex dynamic movement activities (98–101). For instance, in a gymnastics training exercise, gymnasts received an auditory alarm when their hip flexion was >20° (101), and such sonified auditory feedback improved the athletes' hip flexion by 2.3% compared with their baseline performance (101). In another study, an interactive movement sonification system was developed to improve nonvisual motor control while playing a badminton game named “Blindminton,” which was modified for people with vision impairment (102).

Spatially localized vibrotactile feedback has benefits in motion performance, as performance can be guided without the need to provide visual feedback (103) or shift the performer's visual attention (26), which have positive effects regardless of whether the user has impaired vision or not. To maximize the benefits which spatially localized vibrotactile feedback can provide, using vibrotactile arrays composed of multiple tactors can be a potential solution to mitigate the ambiguity observed in the current system. For example, an array of tactors can be worn along or around a body segment to provide linear directional (one-DOF) or rotational guidance (104), or more complex feedback can be enabled using a matrix-shaped (105) or cylindrical design (106) for two- and three-DOF movement guidance.

In previous literature, a combination of multisensory cues was found to improve task performance, accuracy, and spatial attention (35, 83, 84). For specific populations experiencing motor and cognitive function decline (e.g., older adults with age-related degeneration), multimodal feedback improved learning performance and was more strongly preferred (vs. unimodal) during physical tasks (85). From an exercise psychology and instruction perspective, providing multimodal feedback can be viewed as building the “interpersonal relationships” formed in traditional, in-person instruction between instructors and trainees. Given the lack of such interpersonal relationships in remote exercise training platforms, providing personalized and encouraging feedback (107) through multimodal feedback that mimics an in-person instructor's feedback may be effective. The present study provides preliminary evidence that when this relationship is remote, vibrotactile and verbal feedback can improve performance. Future research is needed to explore the degree to which this feedback and “relationship” lead to exercise adherence and long-term engagement.

### 5.5. Methodological contribution

In addition to investigating the effects of sensory feedback on remote yoga training, this study also developed well-defined modular learning scenarios with step-by-step instructions for yoga practice based on a rigorous HTA process, which was an extension of our prior study that introduced only 17 poses (50) compared to the 48 poses included in this study (Supplementary Figures). This is the first study to develop such detailed and realistic modular yoga learning scenarios that take common characteristics of yoga practice sessions into account. We composed each yoga sequence to progress from easier poses to more difficult poses. The connecting arrows (transitions between poses) in Supplementary Figures 1–3 also connect poses with small movement transitions to create smooth and naturalistic pose sequencing, which are common elements in yoga practice.

These learning scenarios may be of particular use in future studies when testing the effectiveness of any new design feature (e.g., feedback modality, feedback design) in a technology-based yoga practice by providing standardized sample pose sequences that are relatively balanced in terms of pose difficulty. They may also help in developing personalized learning modules based on users' physical capabilities. For example, some of the difficult poses (rounded boxes in red color in Supplementary Figures) can be filtered for an individual based on a user profile [e.g., baseline performance measures in terms of balance, range of motion, or strength; (108)] to provide a safe technology-based training environment.

### 5.6. Study limitations

First, the absence of visual demonstrations and feedback during the learning trials may have impacted the NVI participants' overall performance (e.g., the proportion of poses that were correct on the first attempt), particularly as our study recruited novice yogis who were not familiar with the poses typically performed during a yoga session. To isolate the effect of non-visual sensory feedback, our study intentionally eliminated visual demonstrations and feedback, increasing the difficulty and ambiguity of the activity. In general, healthy participants with no preexisting health conditions rely mostly on vision, as vision plays a more significant role than other sensory modalities when learning a physical exercise (109). Given that the target delivery method is remote, providing no visual demonstration was equivalent to or slightly more difficult than using a small screen (e.g., a phone) for remote exercise at home for this participant group (NVI). Thus, in omitting visual demonstrations and feedback from this study, we not only increased the difficulty of the activity, but also improved the study's ecological validity. Similar reasons for not providing visual feedback (rooted in the tendency to rely heavily on visual guidance for exercise training) when assessing the efficacy of vibrotactile feedback can also be found in prior literature (32, 110, 111). Additionally, the results of this study and the observation from the pilot test with the VI subjects revealed possible suggestions and implications for special populations who might have limited visual perception (e.g., people with VI or low visual acuity due to age-related decline) and who need to rely on nonvisual sensory feedback (60).

Second, in this study, movement steps were assessed manually by a trained researcher, rather than automatically by calculating kinematics from the wearable sensors. This manual assessment could have delayed or increased variability in the feedback onset (Fs in Figure 4). As an initial step toward building a remote yoga training platform with personalized, real-time feedback, this study's investigation was focused on testing different feedback types rather than investigating the system's ability to assess motion autonomously. To avoid potential confounding effects caused by this manual assessment, we trained one researcher with practice trials before data collection, and that same person ran every participant session. Furthermore, the potential effect of variation in Fs is minimal in our time measures (FsMe and FeMs), as pose assessment occurs before feedback onset (Fs). In a future study, we could reduce this potential issue by automating the pose assessment process.

Third, the sample size for the VI group was too small to reliably perform certain inferential tests comparing the performance between the NVI and VI groups. This was due to the global pandemic and associated social distancing restrictions, which limited our outreach and access to VI participant recruitment. In future research, we plan to perform a full-scale experiment with a balanced VI participant group to compare performance between NVI and VI groups.

## 6. Conclusions

Effective and time-efficient postural guidance through sensory feedback is critical in designing a remote physical training system for improved performance and user experience. This study investigated the effects of different feedback types (no feedback, verbal alone, vibrotactile alone, and combined verbal and vibrotactile feedback) in a remote yoga training system with 22 healthy adults with normal vision and four adults with impaired vision. Overall, the study found that feedback impacts and enhances the learning and performance outcomes of participants during remote exercise compared to providing no feedback. Regardless of feedback type, additional feedback helped to correct 11.2 and 6.7% poses for the NVI and VI participants, respectively, compared with no feedback condition. The study also found 1–2 feedback events were sufficient in correcting most of the initially incorrect poses. Even though feedback was effective for pose correction, not all feedback types were equally time-efficient. Significantly faster responses were achieved with vibrotactile feedback, which was on average 35.6% faster than verbal only or multimodal (verbal and vibrotactile) feedback. User-reported rankings showed the strongest preferences for Ver+Vib multimodal feedback (57.0%) and verbal feedback alone (33.0%) compared to vibrotactile feedback alone (10.0%) and no feedback (0.0%). A performance comparison between the NVI and VI participants showed similar trends in pose completion rates, except that VI participants recorded a significantly higher proportion of terminal failures and withdrawals for difficult poses. VI participants also had a significantly higher incidence of balance loss compared with NVI participants across all trials.

In summary, this study found that vibrotactile feedback (either as a unimodal or multimodal format combined with verbal feedback) is effective in improving performance during remote physical exercise. In order to increase the effectiveness of vibrotactile feedback in future studies, this study also identified several implications and recommendations, including the use of vibrotactile arrays for providing movement guidance through vibrotactile feedback and movement sonification to decrease the duration of verbal feedback. Through effective multimodal feedback design, it may be possible to provide more accessible options than visual guidance alone for people with diverse functional capabilities. The pilot test with four VI participants confirmed the potential of using our system as a remote exercise option. The investigation also suggested potential implications for design, including sensor attachment location, ways to improve safety of remote exercise, and effective feedback modalities for the target population.

## Data availability statement

The raw data supporting the conclusions of this article will be made available by the authors, without undue reservation.

## Ethics statement

The studies involving human participants were reviewed and approved by the Institutional Review Board, University of Arizona. The patients/participants provided their written informed consent to participate in this study.

## Author contributions

Conceptualization: SLe, SH, and SLi. Data curation: MI. Formal analysis, investigation, methodology, visualization, and writing—original draft: MI and SLi. Funding acquisition, project administration, resources, supervision, and validation: SLi. Software: SLe and SLi. Writing—review and editing: MI, SLe, SH, and SLi. All authors contributed to the article and approved the submitted version.

## Funding

This research was conducted when SLi was an Assistant Professor and MI was a Ph.D. student at the University of Arizona. The research was funded by a faculty seed grant from the University of Arizona. The Open Access Publication was supported by Virginia Tech Open Access Subvention Fund.

## Conflict of interest

The authors declare that the research was conducted in the absence of any commercial or financial relationships that could be construed as a potential conflict of interest.

## Publisher's note

All claims expressed in this article are solely those of the authors and do not necessarily represent those of their affiliated organizations, or those of the publisher, the editors and the reviewers. Any product that may be evaluated in this article, or claim that may be made by its manufacturer, is not guaranteed or endorsed by the publisher.

## Author disclaimer

The opinions expressed in this report are those of the authors and do not necessarily reflect the views of the University of Arizona.
