# Supplementary material for: Effects of vibrotactile feedback on yoga practice
Source: Front Sports Act Living. 2022 Oct 31;4:1005003. doi: 10.3389/fspor.2022.1005003 (PMC9659721; doi:10.3389/fspor.2022.1005003)
Supplement: Supplementary file 1 [file Data_Sheet_1.PDF]

Supplementary Material

1 YOGA POSES AND STEP-BY-STEP VERBAL INSTRUCTIONS USED FOR MODULAR LEARNING

The 48 yoga poses (3 starting poses and 15 pose variations grouped under each starting pose) used in the study, their difficulty levels, the pose sequences used for the learning trials, and the movement steps identified through the HTA process are presented in Figures S1–S6 in this section.

2 SUPPLEMENTARY TABLES AND FIGURES

2.1 Figures

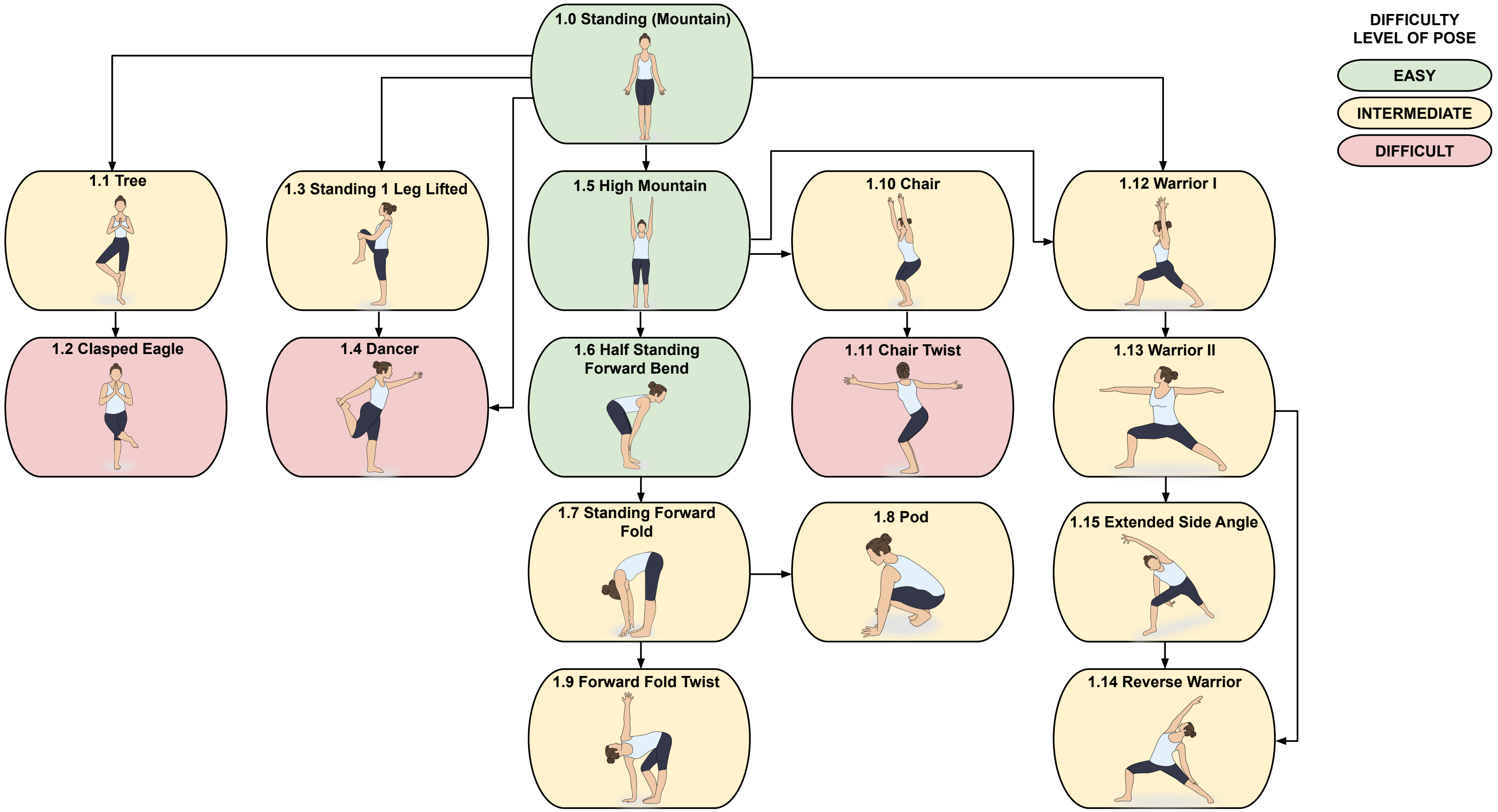

Pose Sequence Used for Each Trial

- 1.0 - 1.12 - 1.13 - 1.14
- 1.0 - 1.12 - 1.13 - 1.15
- 1.0 - 1.5 - 1.6 - 1.7
- 1.0 - 1.4 - 1.0 - 1.1
- 1.0 - 1.12 - 1.13 - 1.15 - 1.14
- 1.0 - 1.5 - 1.6 - 1.7 - 1.9
- 1.0 - 1.5 - 1.6 - 1.7 - 1.8
- 1.0 - 1.12 - 1.0 - 1.1 - 1.2
- 1.0 - 1.1 - 1.12 - 1.0 - 1.3
- 1.0 - 1.5 - 1.12 - 1.13 - 1.14
- 1.0 - 1.5 - 1.10 - 1.11 - 1.0 - 1.4

Figure S1. Standing Pose (1.0) and its variations (1.1–1.15) used in the study. The color of the rounded shape indicates the difficulty of each pose, with green, yellow, and red representing easy, intermediate, and difficult levels, respectively. The possible pose sequences randomly selected for each trial are listed at the bottom.

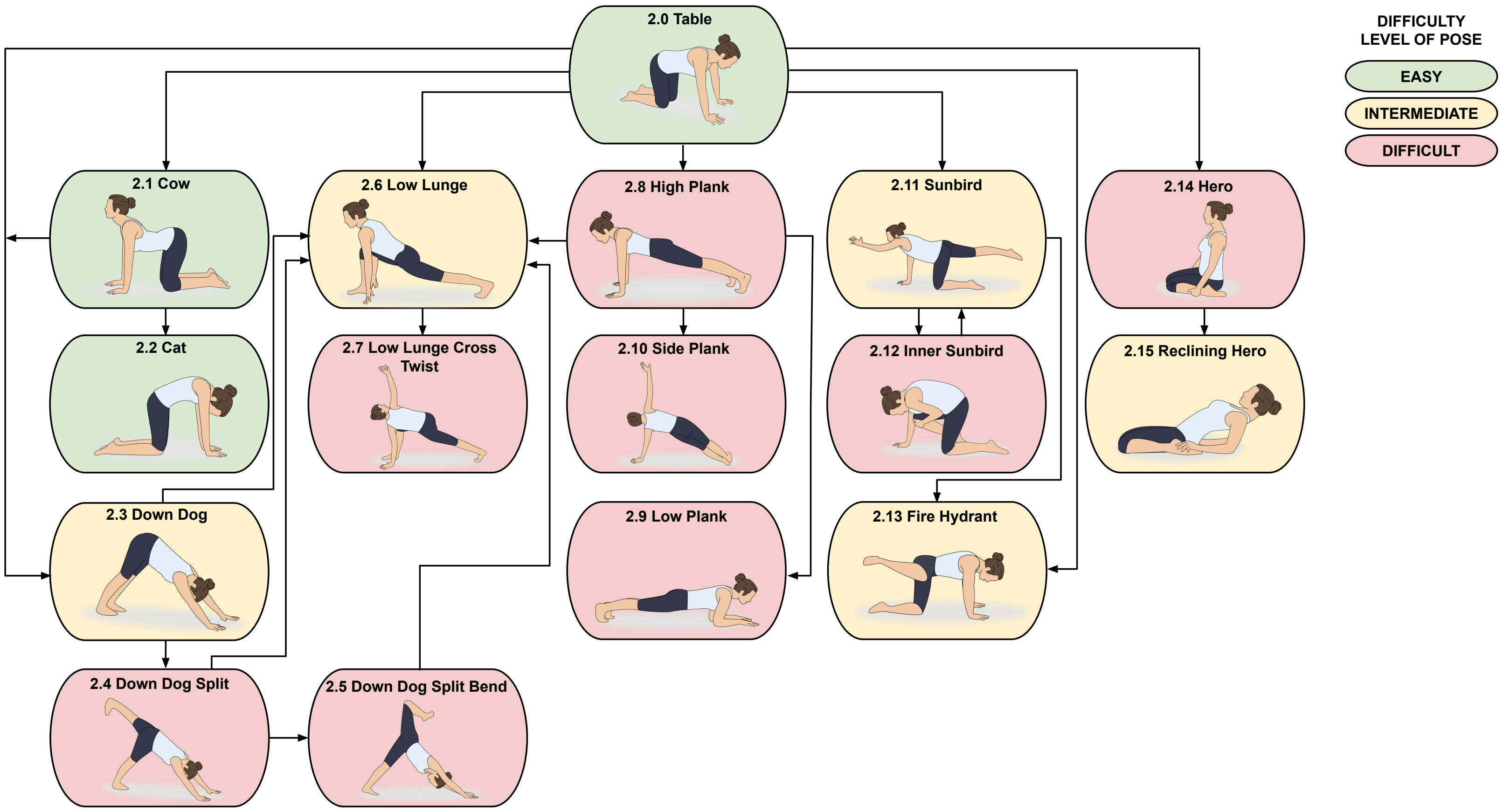

Pose Sequence Used for Each Trial

- 2.0 - 2.3 - 2.4 - 2.5
- 2.0 - 2.11 - 2.13 - 2.0 - 2.1
- 2.0 - 2.8 - 2.6 - 2.7
- 2.0 - 2.11 - 2.13 - 2.0 - 2.8
- 2.0 - 2.3 - 2.6 - 2.7
- 2.0 - 2.11 - 2.12 - 2.0 - 2.13
- 2.0 - 2.14 - 2.0 - 2.13
- 2.0 - 2.8 - 2.9 - 2.0 - 2.14 - 2.15
- 2.0 - 2.8 - 2.10 - 2.0 - 2.11
- 2.0 - 2.14 - 2.15 - 2.0 - 2.1 - 2.2
- 2.0 - 2.3 - 2.4 - 2.6 - 2.7

Figure S2. Table Pose (2.0) and its variations (2.1–2.15) used in the study. The color of the rounded shape indicates the difficulty of each pose, with green, yellow, and red representing easy, intermediate, and difficult levels, respectively. The possible pose sequences randomly selected for each trial are listed at the bottom.

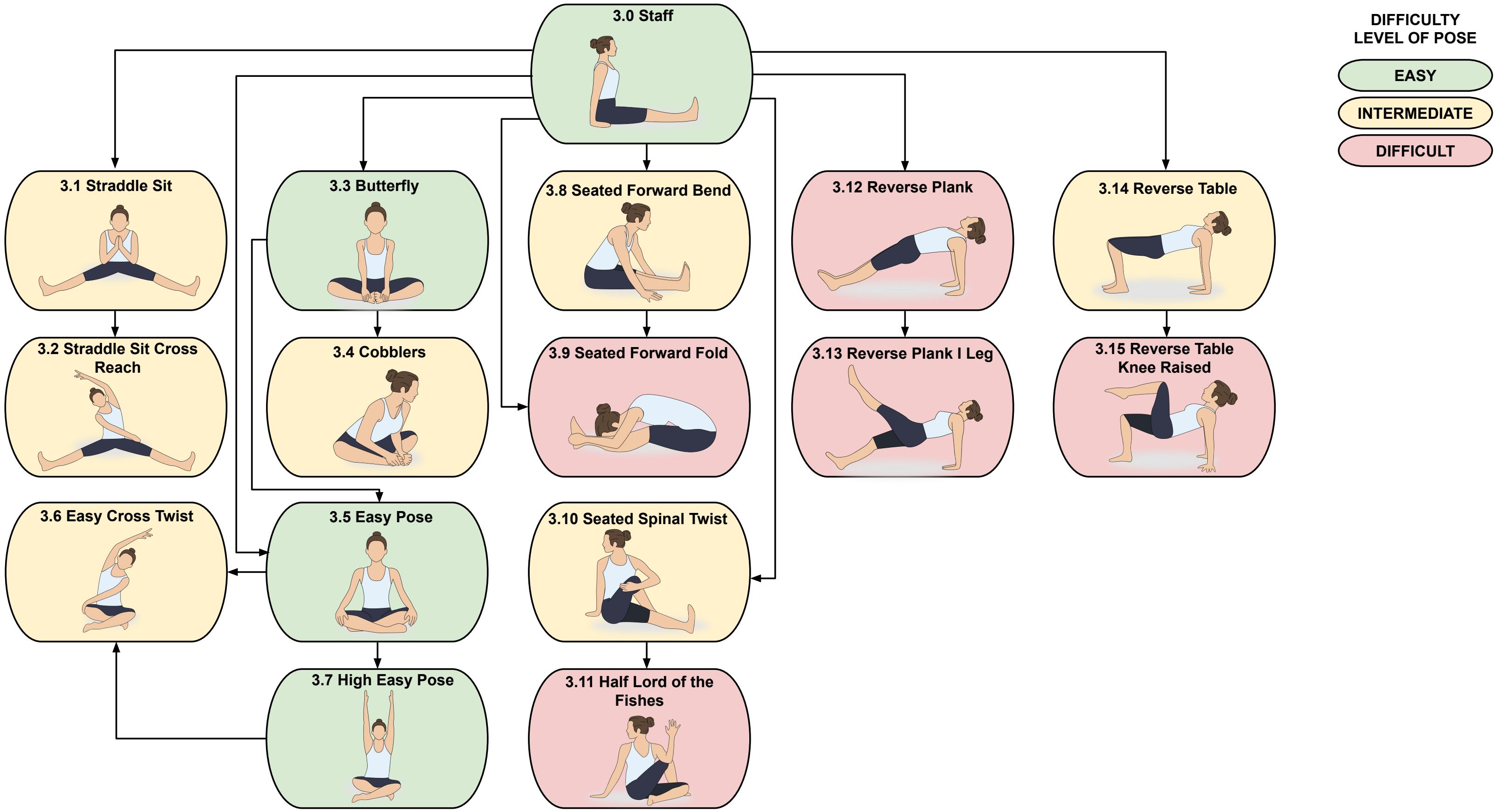

Pose Sequence Used for Each Trial

- 3.0 - 3.14 - 3.0 - 3.12
- 3.0 - 3.10 - 3.0 - 3.12
- 3.0 - 3.5 - 3.7 - 3.6
- 3.0 - 3.1 - 3.2 - 3.0 - 3.12
- 3.0 - 3.3 - 3.4 - 3.0 - 3.9
- 3.0 - 3.14 - 3.15 - 3.0 - 3.10
- 3.0 - 3.3 - 3.0 - 3.8 - 3.9
- 3.0 - 3.1 - 3.2 - 3.0 - 3.10 - 3.11
- 3.0 - 3.12 - 3.13 - 3.0 - 3.8

Figure S3. Staff Pose (3.0) and its variations (3.1–3.15) used in the study. The color of the rounded shape indicates the difficulty of each pose, with green, yellow, and red representing easy, intermediate, and difficult levels, respectively. The possible pose sequences randomly selected for each trial are listed at the bottom.

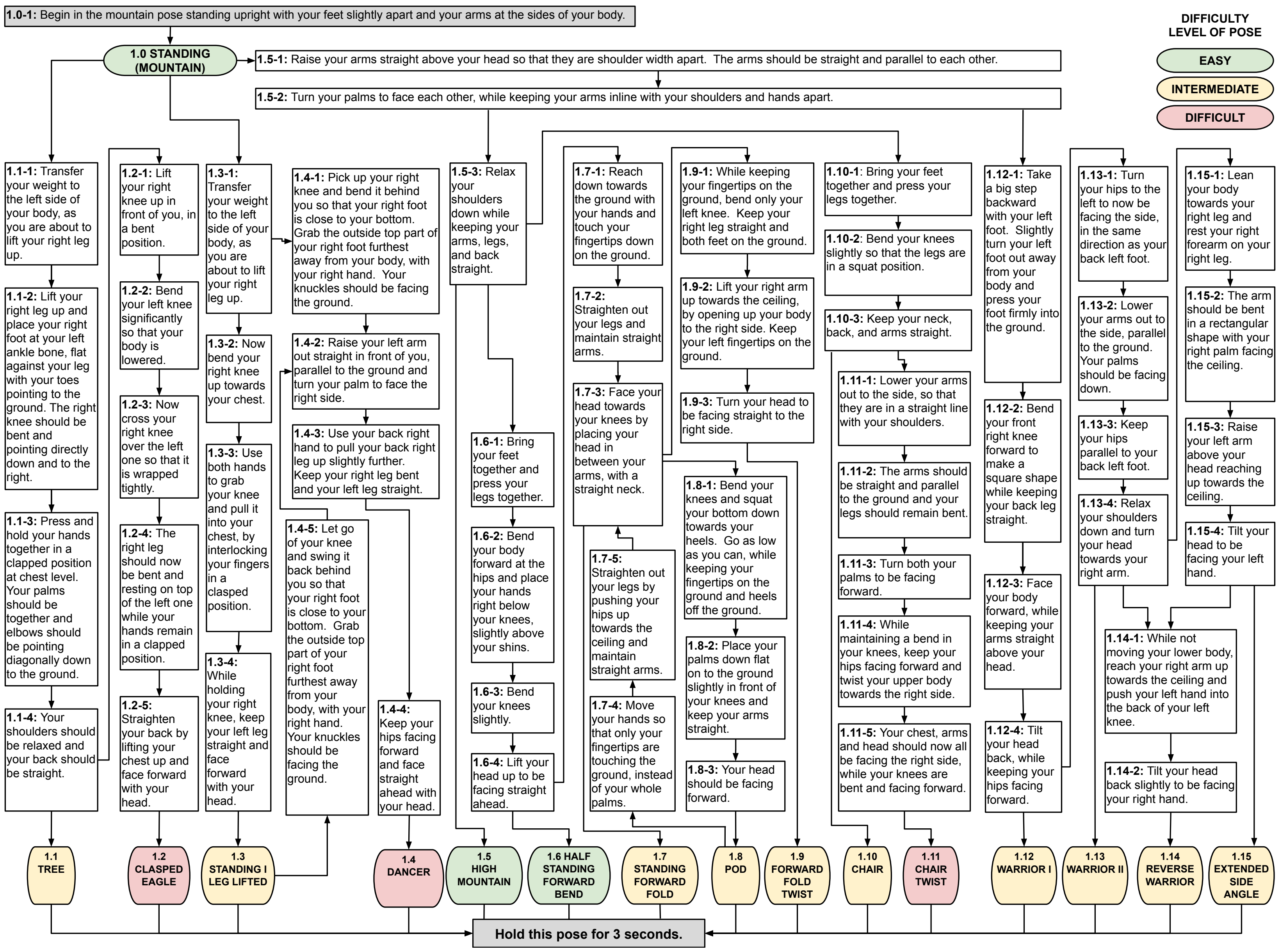

Figure S4. Movement steps for Standing Pose (1.0) and its variations (1.1–1.15) identified through the hierarchical task analysis (HTA). Each rounded shape represents a final yoga pose after following the indicated step-by-step movements (shown as blocks) which were used as verbal instructions in each step. The color of the rounded shape indicates the difficulty of each pose, with green, yellow, and red representing easy, intermediate, and difficult levels, respectively.

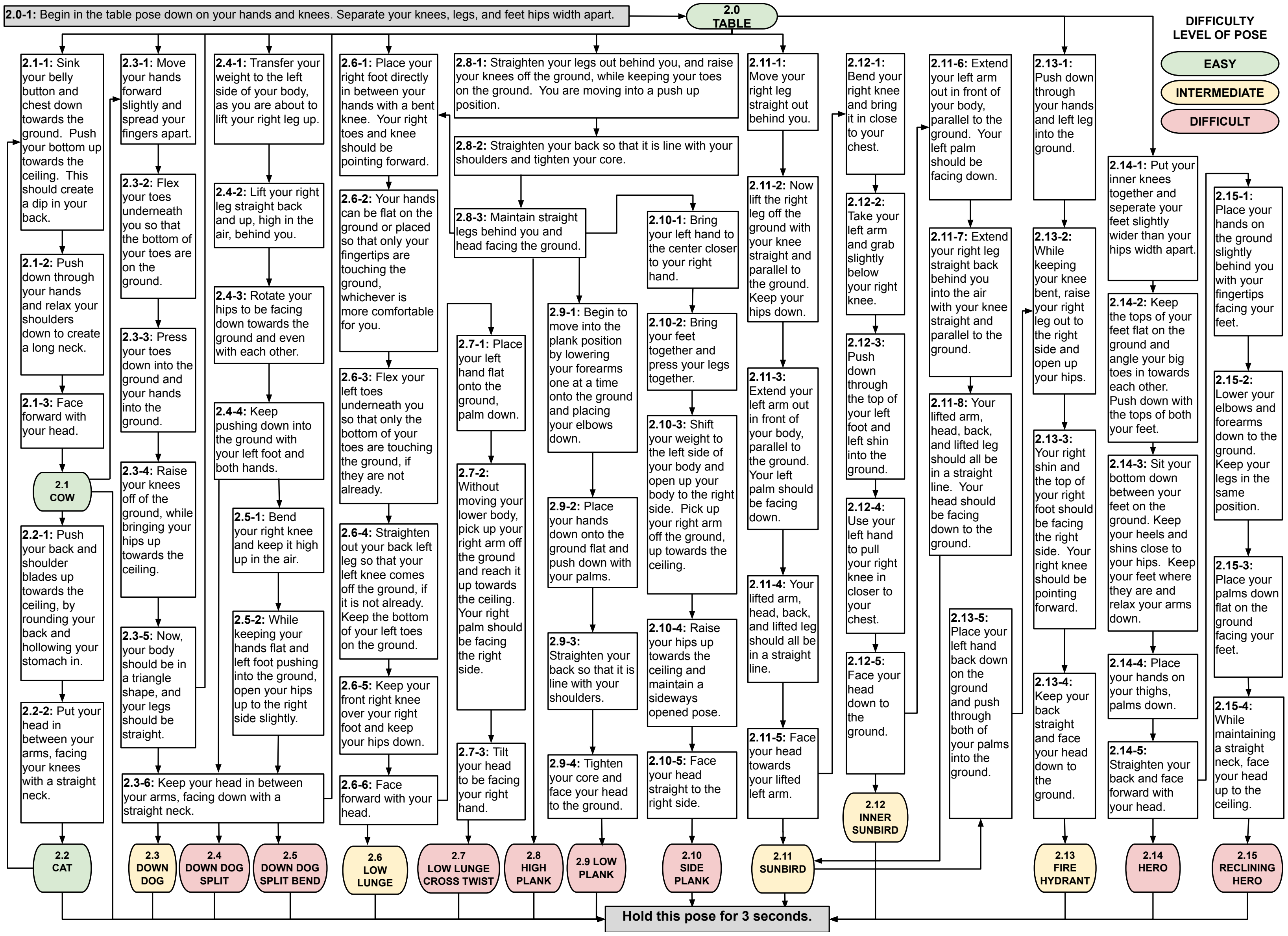

Figure S5. Movement steps for Table Pose (2.0) and its variations (2.1–2.15) identified through the HTA. Each rounded shape represents a final yoga pose after following the indicated step-by-step movements (shown as blocks) which were used as verbal instructions in each step. The color of the rounded shape indicates the difficulty of each pose, with green, yellow, and red representing easy, intermediate, and difficult levels, respectively.

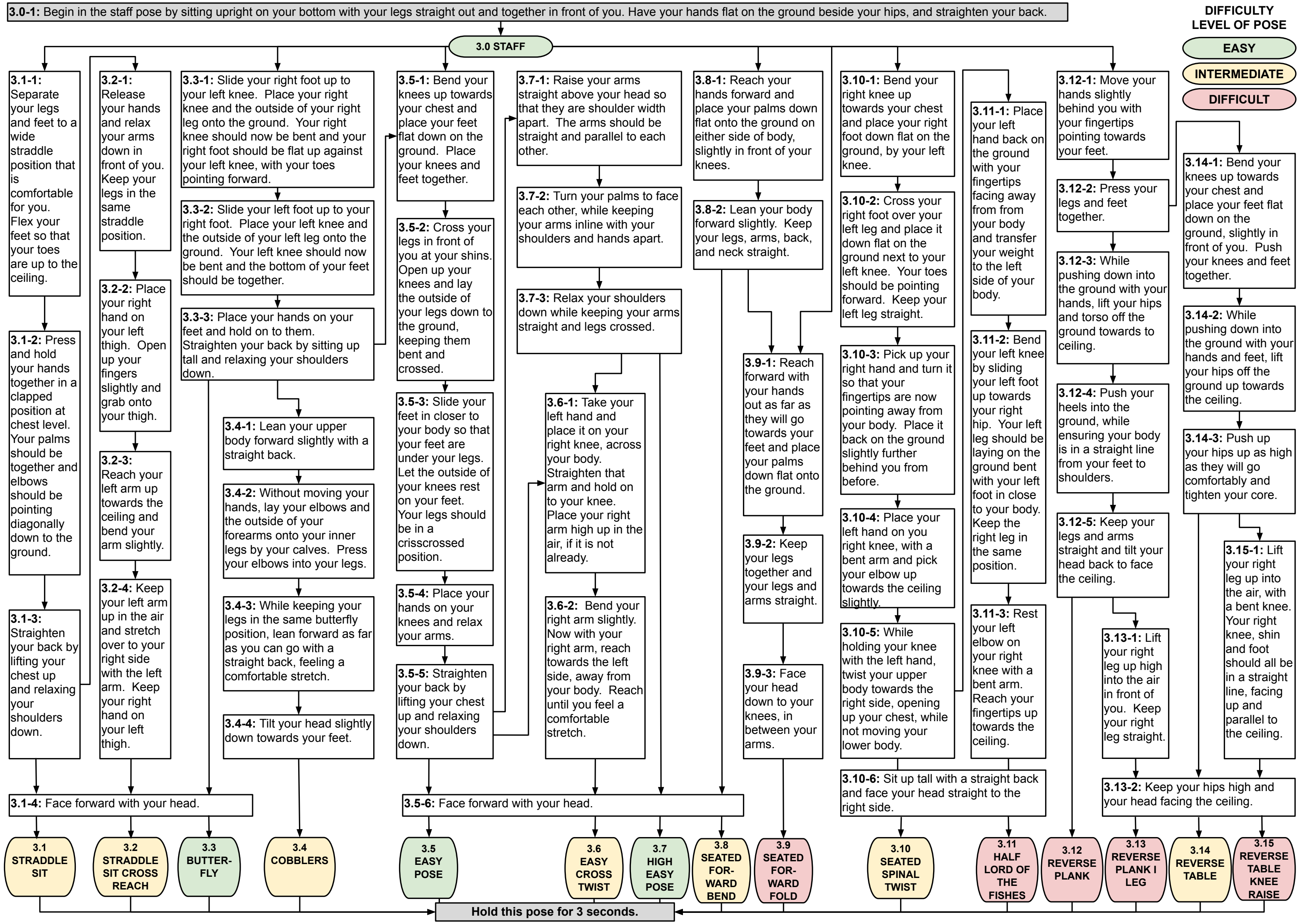

Figure S6. Movement steps for Staff Pose (3.0) and its variations (3.1–3.15) identified through the HTA. Each rounded shape represents a final yoga pose after following the indicated step-by-step movements (shown as blocks) which were used as verbal instructions in each step. The color of the rounded shape indicates the difficulty of each pose, with green, yellow, and red representing easy, intermediate, and difficult levels, respectively.
